# Acquiring Complex Focus-Marking: Finnish 4- to 5-Year-Olds Use Prosody and Word Order in Interaction

**DOI:** 10.3389/fpsyg.2016.01886

**Published:** 2016-12-01

**Authors:** Anja Arnhold, Aoju Chen, Juhani Järvikivi

**Affiliations:** ^1^Department of Linguistics, University of KonstanzKonstanz, Germany; ^2^Department of Linguistics, University of AlbertaEdmonton, AB, Canada; ^3^Department of Languages, Literature and Communication, Utrecht Institute of Linguistics OTS, Utrecht UniversityUtrecht, Netherlands

**Keywords:** information structure, prosody, word order, child language, focus, contrast, givenness

## Abstract

Using a language game to elicit short sentences in various information structural conditions, we found that Finnish 4- to 5-year-olds already exhibit a characteristic interaction between prosody and word order in marking information structure. Providing insights into the acquisition of this complex system of interactions, the production data showed interesting parallels to adult speakers of Finnish on the one hand and to children acquiring other languages on the other hand. Analyzing a total of 571 sentences produced by 16 children, we found that children rarely adjusted input word order, but did systematically avoid marked OVS order in contrastive object focus condition. Focus condition also significantly affected four prosodic parameters, *f*_0_, duration, pauses and voice quality. Differing slightly from effects displayed in adult Finnish speech, the children produced larger *f*_0_ ranges for words in contrastive focus and smaller ones for unfocused words, varied only the duration of object constituents to be longer in focus and shorter in unfocused condition, inserted more pauses before and after focused constituents and systematically modified their use of non-modal voice quality only in utterances with narrow focus. Crucially, these effects were modulated by word order. In contrast to comparable data from children acquiring Germanic languages, the present findings reflect the more central role of word order and of interactions between word order and prosody in marking information structure in Finnish. Thus, the study highlights the role of the target language in determining linguistic development.

## 1. Introduction

To become successful communicators, children need to learn to transmit information in a way that is appropriate for the given context and knowledge state of the interlocutors. For example, answering *Who wants a banana?* with *I WANT a banana* (where capitals indicate a prominent accent) is pragmatically inappropriate and may lead to confusion, even though the sentence itself is morphologically, syntactically and phonologically well-formed and would be perfectly natural in another context. That is, children need to learn appropriate information packaging or information structure marking as part of successful language acquisition. Although languages use different linguistic devices to mark information structure, prosodic marking is often central, as in the use of accentuation in the English example above.

The present study investigates information structure marking in 4− to 5-year-olds acquiring Finnish, a language characterized by employing word order alongside prosody and showing interactions between the two in encoding information structure. We used a language game to gather semi-spontaneous data with different word orders and information structures. Our prosodic analyses revealed effects of information structure on *f*_0_ range, duration and the use of pauses. The study further found significant effects of information structure on voice quality, a dimension that has, to our knowledge, not previously been investigated for information structure marking in child language (but on focus effects in adult speech, Epstein, 2002; Ní Chasaide et al., 2011, for English, Vainio et al., 2010; Arnhold, 2016, for Finnish).

Before turning to the experimental methods in Section 2 and results in Section 3, we will give an overview of basic concepts of information structure that are essential to the current study, existing research on information structure marking in child language and information structure marking in adult Finnish, and state the research questions in the rest of Section 1. We will discuss how the findings relate to the research questions in Section 4 and conclude with Section 5.

The term information structure refers to the way interlocutors organize their utterances to match the common ground, i.e., the information that is shared and known to be shared among them (see, e.g., Krifka, 2008, for an introduction to basic notions information structure). Information structure is often discussed in terms of binary partitions of utterances. Although there is some variation in the literature, scholars generally distinguish between focus and background, between given and new, and between topic and comment. Focus, commonly marked by square brackets and a subscript letter F, is the part of the utterance which indicates that alternatives are relevant for its interpretation, while the rest of the sentence is often referred to as the background (Rooth, 1985, 1992). For example, for the utterance *My [sister]*_F_ *hates broccoli*, with the word *sister* in focus, relevant alternatives include *My brother hates broccoli, My mother hates broccoli*, etc., but not *My sister hates strawberries* or *My sister loves broccoli*. This information structure might appear as an answer to the question *Which of your family members hates broccoli?*, while a different information structure, with the object noun phrase *broccoli* in focus would be induced by a question like *Which vegetable does your sister hate?* These examples contain focus on a single word or noun phrase, but larger constituents and even whole sentences can be focused, e.g., *What's that noise?—[Our neighbors are renovating]*_F_. This distinction is commonly called narrow vs. broad focus following Ladd (1980) (but see Katz and Selkirk, 2011, for an argument against the use of these terms). In this article, we will reserve the use of the term “broad focus” for cases where the whole sentence is in focus and the term “narrow focus” for focus constituents consisting of single words.

A second fundamental distinction is that between given and new, i.e., between denotations that are present in the common ground and those that are not. In fact, this distinction is often characterized as a scale involving for example inferable information in addition (e.g., Gundel et al., 1993, but see for example Schwarzschild 1999, for an account using a binary distinction). In the present materials, all constituents were either mentioned in the immediate context, i.e., clearly given, or were not present in the preceding context at all, i.e., new. Additionally, the given/new distinction and focus/background division were correlated, such that all focused constituents were new and all background constituents were given. While this is frequently the case in naturally occurring discourse as well, the two dimensions are independent in principle, as illustrated by cases like second-occurrence focus (Beaver et al., 2007; Féry and Ishihara, 2009; but see e.g., Lambrecht 1994, for a newness-based definition of focus).

A third important partition distinguishes topic, i.e., what an utterance is about, and comment, i.e., the information given about it (Reinhart, 1981). In English, topics can be marked syntactically for example through fronting, as in the present sentence.

Finally, regarding the notion contrast, focus can be non-contrastive, for example when providing requested information, e.g., *What's the time?—It's [quarter to seven]*_F_ or contrastive (Dik et al., 1981; Gussenhoven, 2008; Krifka, 2008, for more on focus types). A prototypical case of contrastive focus, and the only one to appear in the present materials, is correction, e.g., *Is that your coat?—No, it's [my mother's]*_F_ *coat*. Contrastive topics are possible as well, but did not feature in the present study.

Children's speech shows an influence of information structure even at very early developmental stages, but adult-like ability to mark information structure is attained quite late^1^. Wieman (1976) observed that 2-year-old English-speaking children deviated from their default realization of two-word utterances by accenting words in non-contrastive narrow focus. However, this observation was based on only seven utterances in Wieman's study and did not emerge in a systematic investigation of Dutch-speaking children, who predominantly accented both words (Chen and Fikkert, 2007). Likewise, Behrens and Gut's (2005) case study of a 2-year-old boy acquiring German found that both words were stressed in most two-word utterances. Recently, Grünloh et al. (2015) have suggested that differences between 2- and 3-year-old children and adults in accentuation, particularly young children's failure to de-accentuate given material, is at least partially due to specific characteristics of caregiver speech.

Analyzing descriptions of picture pairs that differed in one feature corresponding to either subject, object or verb in the description, Hornby and Hass (1970) found that English-acquiring 3- to 4-year-olds frequently produced the contrastive constituent with falling accents with wide *f*_0_ ranges, especially for subject constituents. In a similar study, MacWhinney and Bates (1978) reported that the placement of prominent accents on new and focused constituents was already acquired by age three, but its use significantly increased in frequency between age three and age six. However, this finding held most clearly for their English-acquiring participants and to a lesser degree for the children acquiring Italian. By contrast, children learning Hungarian (and Hungarian adults) did not systematically use accents to mark focus, but showed most variation in word order. Note also that focused referents in Hornby and Hass's (1970) and MacWhinney and Bates's (1978) materials were contrastive as well as new. Similarly, Müller et al. (2006) found that German-acquiring 4- to 5-year-olds consistently placed accents on focused constituents with materials in which all focused constituents were contrastive.

Even when patterns of accent placement are overall similar to those of adults, children's speech may still differ in crucial ways. Chen (2011) found that Dutch 4- to 5-year-olds accented foci more frequently than given topics and used a similar set of accents as adults. However, in children's speech focus was less clearly associated with falling accents (see de Ruiter, 2014, for somewhat different results regarding givenness). Chen (2011) only found a completely adult-like use of accent type in 7- to 8-year-olds, but even at this age, focus marking in terms of duration and alignment of *f*_0_ turning points was not yet completely adult-like (Chen, 2009). Wells et al. (2004) have reported that children's ability to mark focus condition prosodically and especially their ability to correctly identify information structure in language input continues to improve between age five and age thirteen. While children underuse *f*_0_ range and duration as markers of focus compared to adults, they employ pause durations more extensively, using longer pauses before focal target words than before non-focal ones (Romøren and Chen, 2015).

Regarding word order, several studies suggest that in contrast to a cross-linguistic tendency to place given before new information in adult language, children aged three to six generally place new before given constituents, although contrasting findings have also been reported (Narasimhan and Dimroth, 2008, and references therein). In a study using the same materials and method as the present study, Sauermann et al. (2011) found no general tendency for either given-before-new or new-before-given order in the productions of German 4-year-olds. Children's productions in this task generally did not reflect information structure effects on word order described for adult language, but it is worth noting that the adult control group exclusively produced unmarked SVO order in response to the same task. Prosodic variation, by contrast, was more extensive and more unified in both groups. Children as well as adults showed significant differences in *f*_0_ and duration between broad focus, narrow non-contrastive and narrow contrastive focus conditions, although not all effects were identical for the two groups. An analogous study on Dutch children yielded very similar results (Chen and Höhle, submitted). Interestingly, prosodic effects were only significant for subject nouns in both languages.

In sum, previous research has indicated crucial development in children's ability to mark information structure between the ages of three and six. It has also provided evidence of cross-linguistic differences in the devices acquired, although most studies so far have focused on children acquiring West Germanic languages.

Finnish marks information structure through syntax, prosody, and, to some degree, morphology. Some clitics like *-kin* “also” mark their host as focused or contrastive, but the role of morphology is least well researched and will not be discussed further here (see Nevis, 1986, for more details).

Syntactically, Finnish is a discourse configurational language, i.e., word order is largely determined by information structure, while grammatical roles are coded through case marking (Vilkuna, 1989, 1995; Vallduví and Vilkuna, 1998). Thus, all constituent permutations are grammatical, but indicate differences in information structure. Table 1 illustrates this, adopting Vilkuna's division of Finnish sentences into the contrast position, the topic position and the rest of the sentence (called K-position, T-position and V-field and identified as Spec(CP), Spec(IP) and I', respectively, by Vilkuna, 1995)^2^. By default, the finite verb is the beginning of the “Rest” and a constituent directly preceding it will be interpreted as topical and/or given, occupying the topic position. Constituents preceding the topic position are generally contrastive and can be topics or foci, whereas non-contrastive foci and new information appear in absolutely final position, at the end of the “Rest”^3^.

**Table 1:** **An example for the connection between word order and information structure in Finnish**.

	**Contrast**	**Topic**	**Rest (focus final)**	**English equivalent**
(a) SVO		*Kissa*	*söi hiiren*	“The cat ate the mouse.”
		cat.nom	ate mouse.acc	
(b) OVS		*Hiiren*	*söi kissa*	“The mouse was eaten by the cat.”
(c) OSV	*Hiiren*	*kissa*	*söi*	“It is the mouse that the cat ate.”
(d) SOV	*Kissa*	*hiiren*	*söi*	“It is the cat that ate the mouse.”
(e) VSO	*Söi*	*kissa*	*hiiren*	“The cat did indeed eat the mouse.”
(f) VOS	*Söi*	*hiiren*	*kissa*	“The mouse was indeed eaten by the cat.”

Unmarked SVO word order is possible with all information structures. Thus sentence (a) in Table 1 is a good answer to different questions like “What happened?” (broad focus), “What did the cat eat?” (narrow focus on the object), and even “Who ate the mouse?” (narrow focus on the subject), even though by default the pre-verbal subject is interpreted as the topic and the final object as being in narrow focus. The OVS sentence in (b), however, is a felicitous answer to “Who ate the mouse?” but not to the other questions. It marks the object as a topic and the subject as non-contrastively focused, a constellation that is sometimes expressed through passivization in English. Finally, the elements in the contrast position are interpreted as contrastive in (c)-(f), so that (d), for example would be an appropriate answer to “Did the dog eat the mouse?” while (e) and (f) can be corrections of claims that the event did not take place (the use of VSO word order (e) is less restricted than that of VOS order (f), see Välimaa-Blum 1988, p. 71; Vilkuna 1995; Jokinen 2005; also note that the use of the contrast position is generally not obligatory and that it is in fact relatively rare in written corpora, as discussed in Section 4.1).

Finnish prosody is affected by information structure in several ways. Finnish *f*_0_ contours normally consists of a series of rise-falls in broad focus, with peaks on all constituents except for finite verbs (Välimaa-Blum, 1993; Iivonen, 1998; Suomi et al., 2008). For constituents in narrow focus, the *f*_0_ range of these rise-falls expands while the *f*_0_ range of the other constituents is compressed (Mixdorff et al., 2002; Vainio and Järvikivi, 2007). The rising-falling shape is not altered by information structural variation, and Finnish is frequently described as having just a single accent, with the same tonal targets and the same alignment realized in virtually all contexts (Välimaa-Blum, 1993; Suomi et al., 2008, but see Arnhold, 2014; Arnhold, submitted, for a different analysis employing phrase tones). Crucially, no account of Finnish prosody has suggested contrasting accents for constituents with different information structural roles. This constitutes a major difference from Germanic languages, where the choice of accent type frequently marks pragmatic distinctions, including information structure. For example, constituents in narrow focus frequently carry falling accents, while contrastive topics are often realized with a rise (e.g., Hedberg and Sosa, 2008, on English and Féry, 1993; Braun, 2006, on German).

In addition to *f*_0_ range adjustments, Finnish prosody is affected by information structure in several ways. Specifically, constituents in narrow focus have longer duration (Mixdorff et al., 2002; Suomi, 2007), higher intensity (Vainio and Järvikivi, 2007; Arnhold, 2016) and are followed by pauses more often than constituents in broad focus (Arnhold, 2016). The prosody of given constituents shows the opposite characteristics, i.e., shorter durations, reduced intensity and absence of following pauses. Further, Vainio et al. (2010) reported a less tense voice quality in narrow focus, while Arnhold (2016) found increased use of different kinds of non-modal voice quality (e.g., creaky voice and whisper) on the second syllables of constituents in narrow focus and all following words in the same sentence.

Finally, prosodic and syntactic marking of information structure interact. For example, Vainio and Järvikivi (2006) found that compared to final words in sentences with unmarked word order, listeners perceived a word that appeared in the sentence-final focus position due to the use of a marked word order as prosodically more prominent, even though both cases were manipulated to have the same prosodic characteristics. Conversely, in production speakers compensated for a mismatch between syntax and information structure by reducing the prosodic prominence of a constituent located in the focus position when a context question implied that another constituent was in narrow focus (Vainio and Järvikivi, 2007). Furthermore, Arnhold and Féry (2013) found that speakers produced more consistent prosodic focus marking in scripted speech with fixed unmarked word order than in semi-spontaneous productions where they were free to use both word order and prosody to mark information structure.

This study investigates children's acquisition of the complex Finnish system of prosodic information structure marking, syntactic information structure marking and interactions between both components. Specifically, it addresses the following questions:

- How do Finnish-acquiring 4- to 5-year-olds use prosody and word order in various focus conditions?
- How do they differ from their peers acquiring West Germanic languages?
- How do they differ from adult Finnish speakers?

Given that children learning Germanic languages attain adult-like information-structure marking quite late, we hypothesize that also children acquiring Finnish differ from adults at ages four to five, and do not yet make full use of all available prosodic and syntactic tools. We further expect that Finnish-acquiring children differ from children learning Germanic languages by showing at least some interactions between word order and prosody in information structure marking, since these interactions are well-attested in adult Finnish speech.

The comparison with adult speakers of the same language on the one hand and children acquiring other languages on the other hand will thereby allow some insight into a further research question: What is the relative importance of universal tendencies and language-specific characteristics for the trajectory of language acquisition?

## 2. Materials and methods

**Figure 1:**
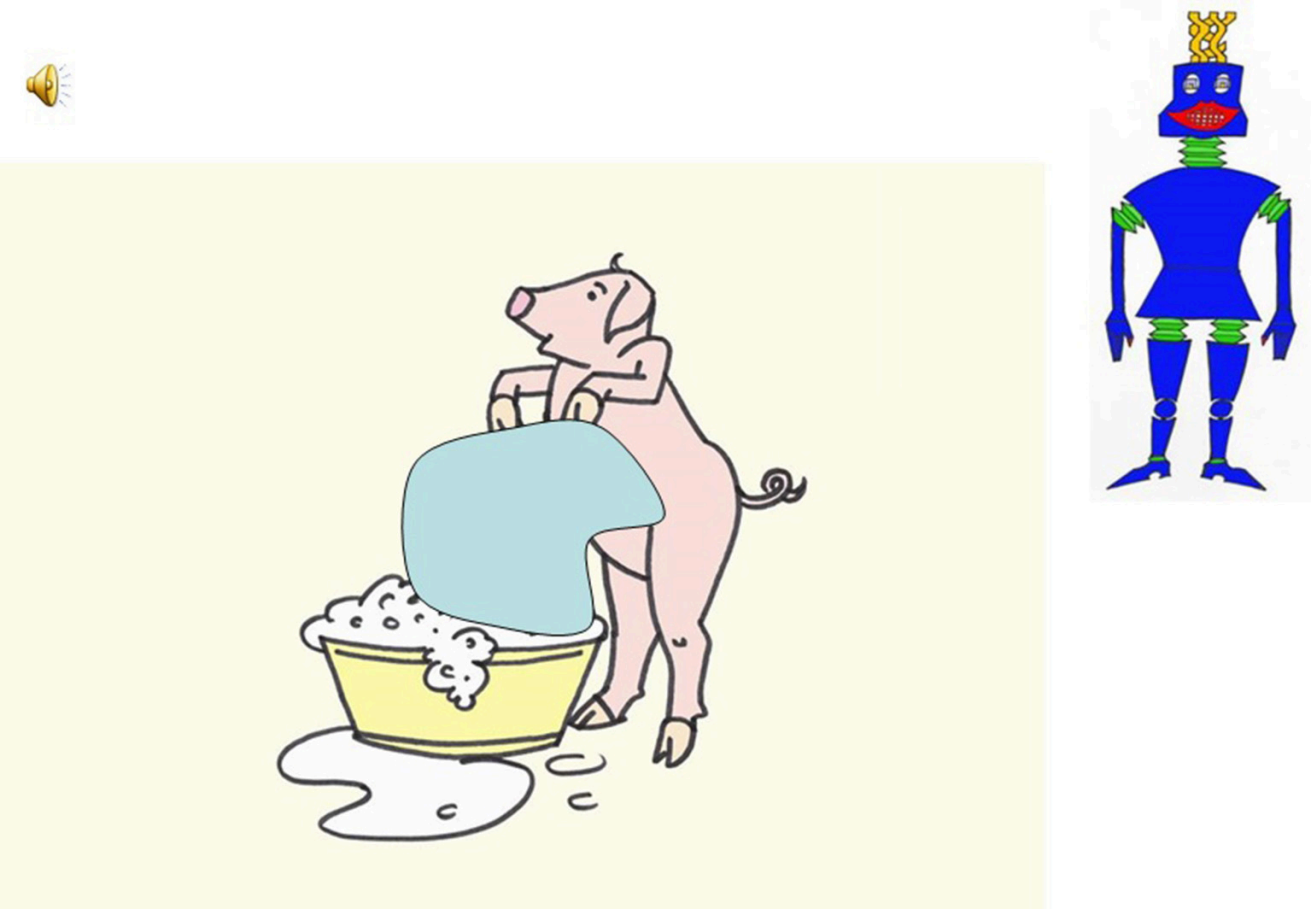
**Stimulus slide for *Possu pesee paitaa* “The piggy is washing a shirt” with non-contrastive object focus (NFO)**.

To elicit short sentences with systematically varied information structures in a controlled way, participants were asked to teach a robot (depicted in Figure 1) to speak Finnish like a human. To this end, the experimenter asked the robot questions about visual stimuli and the children repeated and—if they chose to—adjusted the robot's answers. Crucially, to encourage children to produce sentences with natural information structure marking, the robot's utterances included both contextually inappropriate and appropriate word orders and were produced with unnatural flat *f*_0_ (see below for details). The same method and materials, which allow for controlling information structural conditions in a naturalistic game-type setting, were used in experiments on children acquiring German and Dutch (Sauermann et al., 2011; Chen and Höhle, submitted).

### 2.1. Procedure

Participants were recorded individually in a quiet room at their respective day care facility in Helsinki or Espoo by a female research assistant.

Before the start of the experiment, each participant saw a slide with the picture of the robot, who introduced herself and solicited the participant's help in improving her human language skills. After the procedure was explained to the participant, the introduction concluded with three practice trials.

At the beginning of each trial, the participant saw a slide as illustrated in Figure 1, containing a picture of the robot and visual display of a scene. A part of this scene was covered by a blue shape, but the participant was told that the robot could see the complete scene. The experimenter described the visible part of the scene and then asked the robot about the covered part. For example for the item in Figure 1, the introductory description was *Possu pesee jotain ammeessa* “A piggy is washing something in a tub,” followed by the question *Mitä possu pesee?* “What is the piggy washing?” The robot answered the question, in this case with *Possu pesee paitaa* “The piggy is washing a shirt.” After this, the experimenter posed the same question to the participant, who answered the question based on the robot's answer. The participant's answer thus had the same information structural context as the robot's utterance, i.e., inducing non-contrastive narrow focus on the object *paitaa* “shirt” for the present example. Finally, the experimenter removed the blue cover from the picture to reveal the complete scene. For broad focus items, the central part of the picture was completely covered and the experimenter introduced these items with “Now we cannot see what is happening” or “Now the whole picture is covered again” before asking a broad focus question like shown in Table 2 below.

**Table 2:** **Example question-answer pairs for all experimental conditions**.

	**SVO**		**OVS**	
BF	Q:	Mitä tässä tapahtuu?	Q:	Mitä tässä tapahtuu?
		“What is happening here?”		“What is happening here?”
	A:	[Tyttö lakaisee katua]_F_.	A:	[Paitaa silittää poika]_F_.
		girl.nom sweeps street.prt		shirt.prt irons boy.nom
		“A girl is sweeping a street.”		“A boy is ironing a shirt.”
CFS	Q:	Poimiiko leijona mansikkaa?	Q:	Vetääkö kala autoa?
		“Is a lion picking the strawberry?”		“Is a fish pulling the car?”
	A:	[Varsa]_F_ poimii mansikkaa	A:	Autoa vetää [kameli]_F_.
		foal.nom picks strawberry.prt		car.prt pulls camel.nom
		“A foal is picking the strawberry.”		“A camel is pulling the car.”
CFO	Q:	Peseekö prinsessa autoa?	Q:	Ompeleeko noita nallea?
		“Is the princess washing a car?”		“Is the witch sewing a teddy bear?”
	A:	Prinsessa pesee [maljakkoa]_F_.	A:	[Paitaa]_F_ ompelee noita.
		princess.nom washes vase.prt		shirt.prt sews witch.nom
		“The princess is washing a vase.”		“The witch is sewing a shirt.”
NFS	Q:	Kuka raaputtaa ikkunaa?	Q:	Kuka pitää haarukkaa?
		“Who is scratching the window?”		“Who is holding the fork?”
	A:	[Kameli]_F_ raaputtaa ikkunaa	A:	Haarukkaa pitää [lapsi]_F_.
		camel.nom scratches window.prt		fork.prt holds child.nom
		“A camel is scratching the window.”		“A child is holding the fork.”
NFO	Q:	Mitä isoäiti antaa?	Q:	Mitä lapsi ostaa?
		“What is grandmother giving?”		“What is the child buying?”
	A:	Isoäiti antaa [pallon]_F_.	A:	[Paitaa]_F_ ostaa lapsi.
		grandmother.nom gives ball.acc		shirt.prt buys child.nom
		“Grandmother is giving a ball.”		“The child is buying a shirt.”

*BF, broad focus; CFS, contrastive focus on the subject; CFO, contrastive focus on the object; NFS, narrow focus on the subject; NFO, narrow focus on the object*.

Every participant was presented with 48 experimental items in a pseudo-randomized order. For half of the participants, the presentation order was reversed. Participants' speech was recorded directly onto the hard drive of a laptop computer with a sampling frequency of 44,100 Hz, using a high-quality head-mounted microphone.

### 2.2. Materials

Five different information structural conditions appeared in the robot's as well as the participants' answers: broad focus (BF), contrastive narrow focus on the subject (CFS), contrastive narrow focus on the object (CFO), non-contrastive narrow focus on the subject (NFS), and non-contrastive narrow focus on the object (NFO). For the sake of simplicity, we will refer to contrastive narrow focus as “contrastive focus” and to non-contrastive narrow focus as “narrow focus” in the following. Sixteen trials elicited broad focus, a further 16 trials elicited focus on the subject (8 NFS and 8 CFS), and another 16 trials elicited focus on the object (8 NFO and 8 CFO).

Table 2 shows examples of the experimenter's questions and the robot's answers for all conditions. Note that the information structure of the (robot's and participants') answers was not only set up in the experimenter's question, but also in her preceding description of the the picture, as well as in the picture itself. Thus, for broad focus, the whole scene was covered in the picture and the experimenter introduced the trial by stating something like *The whole picture is covered* or *The picture is again covered so that we cannot see what is happening*. By contrast, in all contrastive and non-contrastive narrow focus conditions, only the character who was supposed to be focused in the answer was covered in the picture (e.g., the shirt in Figure 1). The experimenter's introductions of these trials accordingly replaced the hidden entity with *someone* or *something*, while naming the other entity and the action (see above for the introductory description for Figure 1, which mentions that the piggy is washing something). Thus, the named entities can be treated as given and the focused entity as focused in the answer not only because of the question, but also based on the previous conversational turn. For the contrastive focus condition, the context first set up the narrow focus the same way before introducing the contrast between the incorrect replacement for *someone/something* suggested in the experimenter's question and the correct replacement given in the robot's answer (see Table 2) and revealed when the blue cover was removed from the picture.

The second condition varied in the experiment was the word order of the robot's utterance; she produced 24 sentences in SVO order and another 24 in OVS order. Recall that both word orders are grammatical in Finnish, but while SVO is the unmarked order, OVS is not always information-structurally appropriate. We restricted our attention to SVO and OVS, since these word orders are more frequent than word orders involving the contrast position (Hakulinen and Karlsson, 1995, see discussion in Section 4.1) and it can thus be assumed that children acquire them earlier. Furthermore, this restriction reduced the complexity of the experiment and allowed for better comparability to parallel studies on Dutch and German (Sauermann et al., 2011; Chen and Höhle, submitted).

Both factors were crossed as illustrated in Table 2. The item list thus consisted of 48 sentences. All sentences were unique, but most of the subject and object words appeared in more than one sentence, although always in a different information structural condition and frequently combined with a different verb and a different object or subject, respectively (see for example the subject *kameli* “camel” in conditions NFS-SVO and CFS-OVS and the object *paitaa* “shirt” in the conditions BF-OVS, CFO-OVS, and NFO-OVS in Table 2). Subjects were always in nominative case, while objects carried partitive or accusative case, depending on the verb. The complete list of question prompts and robot answers is available as Supplementary Material.

All robot utterances were created from words spoken by a 24-year-old female native speaker of Finnish from Helsinki. To create stimuli devoid of natural sentence-level prosody, the speaker first recorded all words separately in random order. Second, flat *f*_0_ of around 200 Hz was imposed for all words, using the program Praat (Boersma and Weenink, 1992–2014). The temporal structure of the words was preserved, since Finnish has lexical quantity distinctions, so that manipulating segment durations could impact perceptibility. Finally, the words were concatenated into sentences, with 200 ms pauses between all words, as well as at the beginning and end of each sentence. As mentioned above, the robot's utterances included pragmatically inappropriate word orders in addition to this unnatural prosody. To further encourage the participants in their role as language teachers, the robot's introduction before the beginning of the experiment contained morphological agreement errors. The experimental prompts, i.e., the robot's answers to the experimenter's questions, however, did not contain this type of error to minimize task load.

### 2.3. Participants

Twenty four- to five-year-old Finnish children from the Helsinki area participated in the study (10 male and 10 female, mean age: 5;1, range: 4;6–5;6). One participant was excluded from the analysis because the daycare teacher expressed concerns regarding language development, three other participants were excluded because they had trouble following the experimental protocol. Thus, we analyzed data from 16 participants.

Dates of birth were not recorded for three of the participants in the final data set. Their data were retained as they were confirmed to be within the target age range. Alternative subset analyses excluding these three participants were conducted for all dependent measures. They confirmed the same result patterns as the analyses reported below, although some effects were weakened as is to be expected when noticeably reducing the number of data points. Since one of the three participants without exact age information also reported to speak English, we performed further subset analyses excluding only his data, which only showed different significance levels for one of the measures, i.e., the use of pauses after sentence-medial verbs, but otherwise returned the same results as the models of the complete data set. We therefore report the results from the complete data set.

The experiments of the present study were non-invasive and were carried out in accordance with Finnish law and adhered to the guidelines of the Declaration of Helsinki, the American Psychological Association, and the ethical policies of the University of Helsinki. The university abides by the guidelines of the Finnish Advisory Board on Research Integrity on the responsible conduct of research and procedures for handling allegations of misconduct as well as on ethical review in human sciences. As we obtained parental consent for the participants' research participation, this study was exempt from ethics review and approval by the University of Helsinki Ethical Review Board in the Humanities and Social and Behavioral Sciences.

## 3. Results

We removed responses from trials that were unsuitable for analysis, for example because the experimenter had asked the wrong question, inducing a different information structure than intended, because the participant imitated the robot and spoke with flat *f*_0_ or because the participant gave an elliptical one-word answer. Altogether, we excluded 26% of trials, retaining 571 sentences for analysis^4^.

We analyzed the data with respect to *f*_0_ range, duration, pauses and voice quality, because effects of information structure on those measurements have been found in Finnish adults. We additionally evaluated choice of word order in the participants' utterances. Intensity was not analyzed since recording quality varied too widely to allow reliable measurements, despite the use of a head-mounted microphone.

The analyses of all the above-mentioned dependent variables were performed using linear mixed-effect models in R (Baayen, 2008; R Core Team, 2015), as implemented in the package lme4 (Bates et al., 2015). These models test the significance of predictor variables, as well as including random effects to model for example participant variation in the data (Baayen et al., 2008). Here, for the analysis of word order produced by our participants we tested information structure induced by the experimenter's question (levels: BF, NFO, NFS, CFO, CFS) and word order (levels: SVO, OVS) as predictor variables (fixed effects). For the prosodic analyses, since measures were obtained for individual words, we additionally included the predictor constituent, which coded the grammatical role of the word (levels: subject, object). As random effects, we tested participant and lexical item. We determined the model with the best fit to the data by comparing the log likelihood of models including different variables with the anova function. Only variables significantly contributing to an improved model fit were retained. *P*- and χ^2^ values for the significance of predictors were obtained from these model comparisons. For models of categorical dependent variables (here: word order, voice quality, and occurrence of pauses), which were binomial (also see Jaeger, 2008), which were binomial, the model output included *p*-values indicating the significance of differences between factor levels. For models of continuous variables (here: *f*_0_ range, duration, and pause duration), *p*-values were obtained with the package lmerTest (Kuznetsova et al., 2015).

### 3.1. Word order

**Figure 2:**
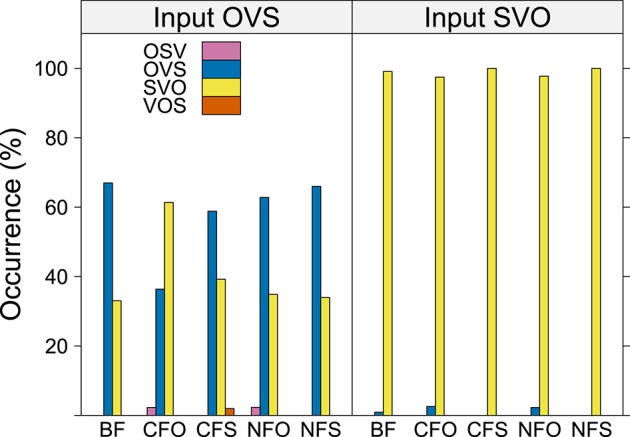
**Word order of participants' utterances by information structure for input word order OVS and SVO**.

Participants used SVO word order in 68% of their utterances and OVS order in 32% of their utterances (388 and 180 cases, respectively), while they used OSV and VOS word order in less than one percent of the entire data set. Since the number of sentences produced with other word orders was negligible, we conducted statistical modeling for SVO vs. OVS responses with binomial models. Model comparison suggested that word order in the participants' utterances (“output word order”) was significantly affected both by the word order used by the robot (“input word order”) and by the information structure induced by the experimenter's question [*p* < 0.001, χ^2^ = 327.4 and *p* = 0.006, χ^2^ = 14.5, respectively]. A more complex model including an interaction between the two factors did not converge for this data set, i.e., there were not enough data points to compute a reliable interaction model. The best linear mixed-effects model of output word order indicated that participants produced SVO order significantly more frequently in response to SVO input word order than in response to OVS input word order [*estimate* = 5.7509, *SE* = 0.6346, *z* = 9.0617, *p* < 0.001; positive estimates indicate more, negative ones less SVO productions]. When the input word order was unmarked SVO, participants also produced SVO utterances in 99% of the cases, with almost no difference between the information structures (see the right panel of Figure 2). Only one OVS utterance appeared in broad focus, contrastive object focus and narrow object focus each. When the robot's utterance used OVS, participants also retained the input word order in the majority of cases (60% overall).

However, the model also revealed an effect of information structural conditions: Compared to the broad focus condition, participants uttered significantly more SVO sentences only in the contrastive object focus condition [*estimate* = 1.5129, *SE* = 0.4316, *z* = 93.5054, *p* < 0.001]. As can be seen in the left panel of Figure 2, participants produced about a third of the input OVS sentences with an output SVO order in most information structural conditions, but realized 61% sentences of input OVS sentences with output SVO order in contrastive object focus. Even though the interaction between word order and focus condition could not be tested for the data set as a whole, the figure thus strongly indicates that the significant difference between the conditions BF and CFO stems from sentences with input OVS word order. To assess this, we created a subset of the data and performed a linear mixed-effects analysis only for the two critical information structural conditions. Modeling results were inconclusive, but provided some limited evidence for an interaction^5^. Note that in the condition combining input OVS order with CFO focus condition, the robot's production constituted a mismatch between the information structure induced by the experimenter's question (contrastive object focus) and the information structure implied by the word order (narrow subject focus). Participants almost never corrected this mismatch with an explicit syntactic marking of contrastive object focus, i.e., by placing the object in the sentence-initial contrast position (Table 1 above). A possible interpretation is that at age five, Finnish children have not yet acquired the use of the sentence-initial position for marking contrast. Instead, they frequently realized SVO word order and placed the contrastive object sentence-finally, in the default position for narrow focus. Recall, however, that as the unmarked word order, SVO is felicitous in most information structural contexts. Therefore, substituting it for the more marked OVS order achieved a better alignment between syntax and information structure for contrastive object focus. However, this happened only for contrastive object focus condition, whereas no significant difference from broad focus was observed for the other information structural conditions, including narrow object focus.

To sum up, participants were strongly influenced by the word order of the input and overwhelmingly used SVO and OVS sentences. Less than 1% of their utterances had a filled contrast position. Notably, only one significant difference appeared between information structural conditions, and there was some limited evidence that it stemmed from sentences with input OVS word order: Participants used the unmarked SVO order more often in contrastive object focus than in broad focus, placing the focused constituent in sentence-final position.

### 3.2. F_0_

**Figure 3:**
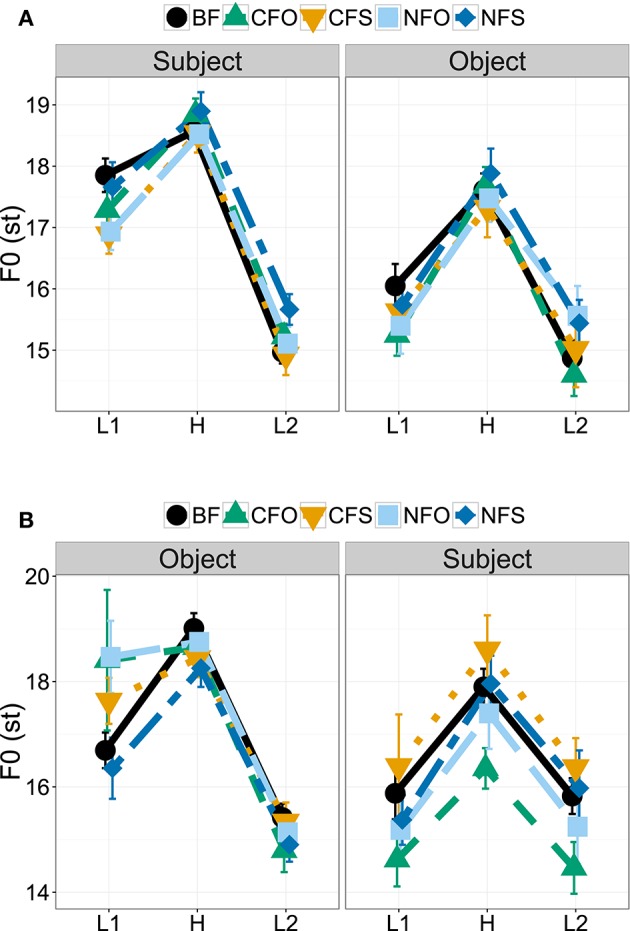
**Mean *f*_0_ measurements and standard errors for SVO (A)** and OVS word order **(B)**.

**Figure 4:**
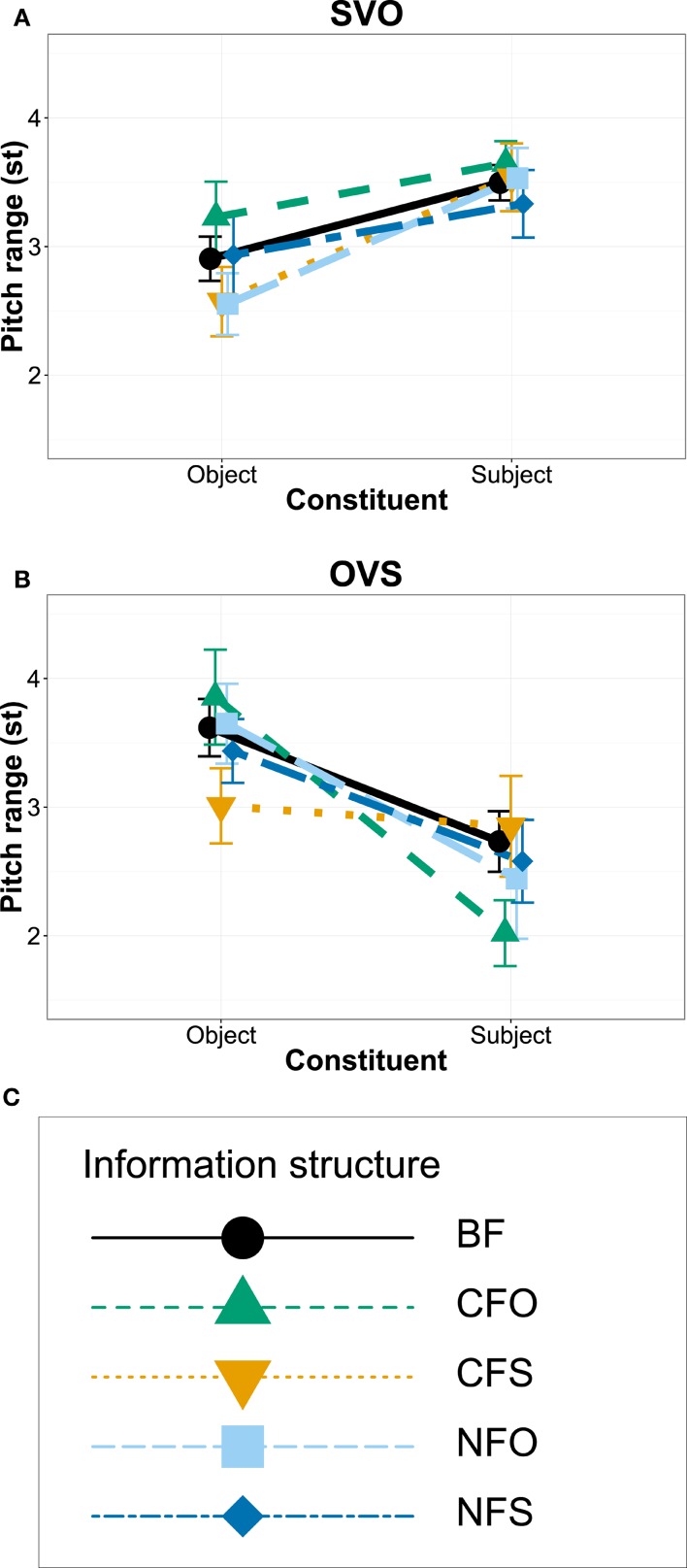
**Interaction plots showing means and standard errors for f0 range of subject and object constituents in SVO word order (A)**, in OVS word order **(B)**, and legend for coding of information structural conditions with line type, plot symbols and color **(C)**.

We analyzed prosody for the 568 sentences that the participants produced with either SVO or OVS order. Out of the 1136 subject and object nouns, 42 subjects and 53 objects could not be analyzed with respect to their *f*_0_ due to bad sound quality or the presence of non-modal voice quality (see Section 3.5). Thus, we analyzed the *f*_0_ of 1041 words. Figure 3 plots the average values of three pitch measurement points for subject and object nouns in different information structural conditions for both word orders: the *f*_0_ maximum (H) and the minimum before (L1) and after it (L2) within the same word. Figure 4 directly compares *f*_0_ range in different conditions (calculated as the distance between the maximum H and the lower one of the two minima). All measurements were converted to semitones (st) relative to a reference value of 100 Hz and statistical analyses evaluated *f*_0_ range.

The best linear mixed-effects model included a significant interaction between word order and constituent [*p* < 0.001, χ^2^ = 44.9]. It suggested that *f*_0_ ranges decreased over the course of the utterance, i.e., *f*_0_ ranges were larger for objects than for subjects in OVS sentences and larger for subjects than for objects in SVO sentences, as illustrated in Figures 3, 4. Accordingly, the model contained significant main effects indicating overall smaller *f*_0_ ranges for subject constituents than for objects [*estimate* = −0.9148, *SE* = 0.1927, *t* = −4.747, *p* < 0.001] and smaller *f*_0_ ranges in SVO output order than for the OVS intercept [*estimate* = −0.7711, *SE* = 0.1629, *t* = −4.735, *p* < 0.001], together with an interaction suggesting that subjects in SVO sentences had larger *f*_0_ ranges [*estimate* = 1.5768, *SE* = 0.2326, *t* = 6.778, *p* < 0.001]. Figures 3, 4 show a clear peak downstep and, as a result, a reduced *f*_0_ range on the sentence-final constituent in both word orders.

There was little indication of a significant effect of information structure for the data set as a whole, as adding this predictor did not significantly improve model fit [*p* = 0.348, χ^2^ = 4.5]. A model including an interaction between information structure and constituent likewise did not provide a significantly better fit [*p* = 0.315, χ^2^ = 9.3], even though it included significant interactions and effects of information structure indicating that *f*_0_ range was marginally larger in broad focus than for contrastive subject focus [*estimate* = 0.4606, *SE* = 0.2396, *t* = 1.922, *p* = 0.056] and significantly larger in contrastive object focus than in contrastive subject focus [*estimate* = 0.7713, *SE* = 0.2865, *t* = 2.692, *p* = 0.008], while the difference between contrastive subject focus and contrastive object focus was significantly smaller for subjects than for objects [*estimate* = −0.8893, *SE* = 0.4074, *t* = −2.183, *p* = 0.030] and the difference between contrastive and narrow subject focus was marginally smaller for subjects than for objects [*estimate* = −0.6646, *SE* = 0.3840, *t* = −1.731, *p* = 0.084].

Figures 3, 4 illustrate that the largest differences between the conditions CFO and CFS appeared in OVS word order, while lines representing different focus conditions in SVO order overlap in Figure 3A. Indeed, a subset model of SVO sentences suggested no significant effect of information structure [*p* = 0.486, χ^2^ = 3.4], only effects of an *f*_0_ downtrend over the course of the sentence, i.e., a significant difference between subjects and objects [*p* < 0.001, χ^2^ = 24.9], with larger *f*_0_ ranges for the initial subjects [*estimate* = 0.6634, *SE* = 0.1317, *t* = 5.038, *p* < 0.001]. For OVS order, by contrast, Figures 3B, 4B display lower *f*_0_ maxima (H) and smaller ranges of the sentence-final subjects in contrastive object focus condition, but raised maxima and larger ranges when the subjects themselves were contrastively focused. To test these differences in the face of contradictory evidence from modeling the whole data set, we conducted subset analyses for the two contrastive focus conditions in OVS order. Here, the best model contained an effect suggesting overall marginally smaller *f*_0_ ranges for subject focus (CFS) than for object focus (CFO) [*estimate* = −0.8707, *SE* = 0.4462, *t* = −1.951, *p* = 0.055] and significantly smaller *f*_0_ ranges for subjects than for objects [*estimate* = −1.9552, *SE* = 0.5470, *t* = −3.574, *p* < 0.001], in addition to significant interaction between constituent and information structure [*p* = 0.01, χ^2^ = 6.7; *estimate* = 1.7852, *SE* = 0.6730, *t* = 2.653, *p* = 0.01]. This suggests that the *f*_0_ range of contrastively focused constituents was significantly extended in this word order, while the *f*_0_ range of unfocused constituents was compressed at the same time.

In summary, there was some evidence that participants' pitch scaling differed between the two output word orders: In sentences that the participants produced with SVO word order, there was no significant effect of information structure. In OVS sentences, by contrast, a subset model suggested that *f*_0_ range was expanded when the constituent itself was contrastively focused, but was compressed for contrastive focus on the other constituent.

### 3.3. Duration

We measured the duration of all subjects and objects in the data set and analyzed subjects and objects in the 568 sentences with SVO and OVS output order (1136 items). The length of target words varied in terms of number of syllables and segments, so that evaluating total word duration was not informative. Therefore, we divided total word duration by number of syllables and evaluated the resulting measure of mean syllable duration. Although Finnish is a quantity language with some isochronous tendencies (Iivonen, 1974, found longer phoneme durations in shorter words, Suomi and Ylitalo, 2003, found no tendency for syllable isochrony, Suomi and Ylitalo, 2004, observed isochronous tendencies for disyllabic feet, but not for longer ones), this considerably reduced variability between items in size, diminishing overall standard deviation from 196 ms for words to 79 ms for syllables.

**Figure 5:**
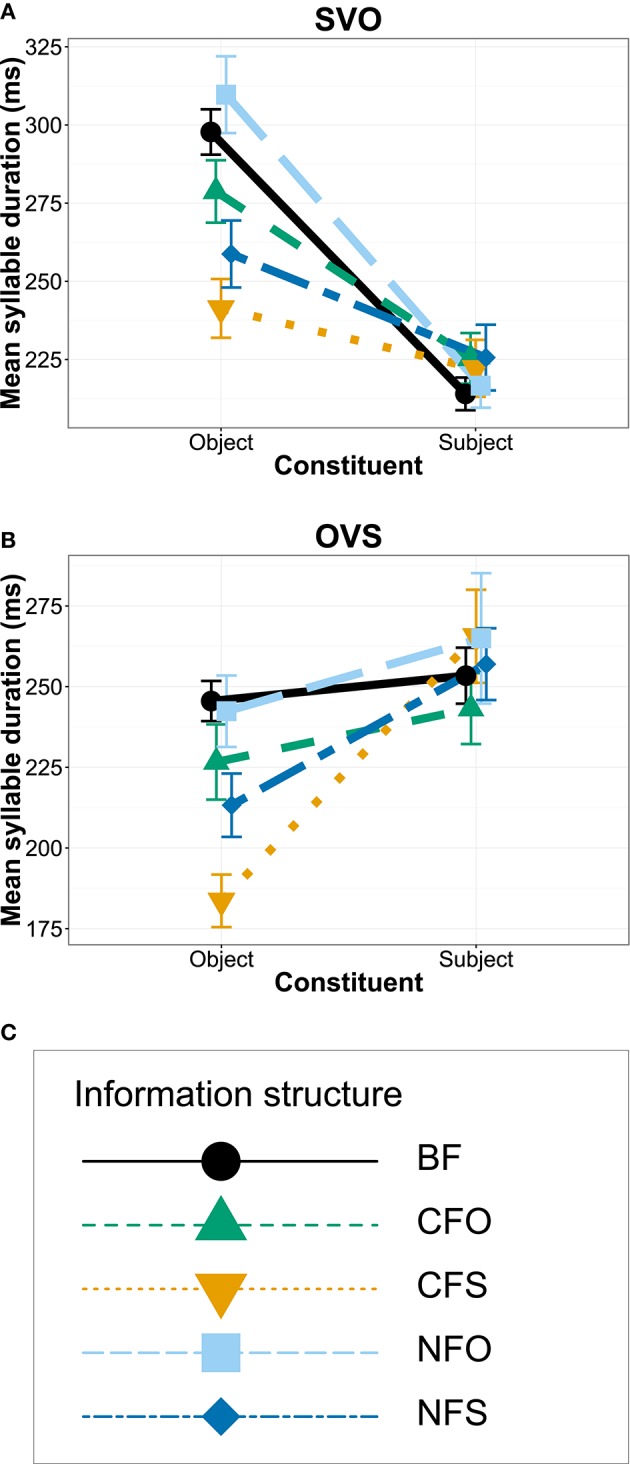
**Interaction plots for mean syllable duration of subject and object constituents in different information structural conditions in SVO word order (A)**, in OVS word order **(B)**, and legend for coding of information structural conditions with line type, plot symbols and color **(C)**.

Statistical modeling of the data suggested significant final lengthening, i.e., longer durations for constituents in sentence-final position: The model indicated shorter syllable durations in output SVO order than in OVS order [*estimate* = −36.9634, *SE* = 5.9302, *t* = −6.2331, *p* < 0.001] and a positive interaction between the factors constituent and word order suggested that durations were longer in SVO word order for objects than subjects [*p* < 0.001, χ^2^ = 128.8; *estimate* = 97.1528, *SE* = 8.3127, *t* = 11.6873, *p* < 0.001]. Thus, only final objects had longer durations in SVO order, while initial subject durations were shorter. This is illustrated in Figure 5A, which shows mean syllable duration in the different information structural conditions. By contrast, Figure 5B displays generally longer durations for subjects in OVS word order. While the main effects of information structure were not significant, the best model contained a significant interaction indicating an effect of information structure on object durations [*p* = 0.012, χ^2^ = 12.9]: Durations of object nouns were significantly shorter in both conditions with narrow focus on the subjects (CFS and NFS) than in broad focus condition. The effect was larger for contrastive focus, but significant for narrow focus as well [*estimate* = −59.9199, *SE* = 17.9352, *t* = −3.3409, *p* = 0.001 and *estimate* = −40.3638, *SE* = 17.4382, *t* = −2.315, *p* = 0.023, respectively]. Adding an interaction between information structure and word order or a three-way interaction between information structure, word order and constituent (subject vs. object) did not improve the model significantly [*p* = 0.825, χ^2^ = 1.5 and *p* = 0.819, χ^2^ = 4.4, respectively], indicating that the effect of information structure did not differ between word orders. The analysis thus indicated a consistent strategy of shortening object durations in subject focus conditions for both SVO order, where it curtailed final lengthening, and OVS order, where the shortened object was in topic position (see the shorter durations for objects for conditions NFS and CFS in Figure 5).

Thus, this section presented information structure effects on the duration of objects in both word orders—SVO and OVS—and for both focus types—contrastive or narrow focus. Object nouns showed significantly reduced mean syllable durations when subject nouns were in focus.

### 3.4. Pauses

**Figure 6:**
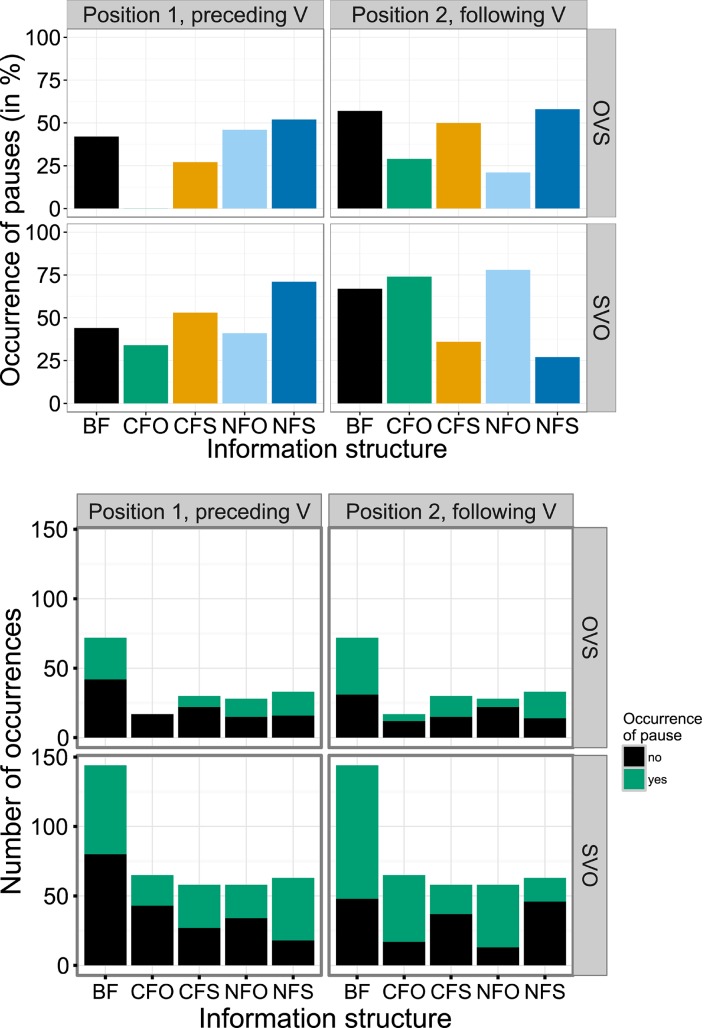
**Occurrence of pauses before the verb (position 1) and after the verb (position 2) for OVS and SVO sentences in different information structural conditions in percent (A)** and in absolute numbers **(B)**.

There are two relevant positions for evaluating the occurrence of pauses, defined as a perceivable silence irrespective of duration (see below), in the short SVO and OVS sentences analyzed here: first, before the verb, i.e., after the subject in SVO sentences and after the object in OVS sentences, and second, after the verb, i.e., before the object in SVO sentences and before the subject in OVS sentences. As is common in child data (Redford, 2013), pauses were frequent in our participants' productions. Altogether, 567 pauses appeared in the 568 sentences evaluated here, of which 45% preceded the verb and 55% followed the verb. Figure 6 shows their distribution by information structural condition and output word order.

The best binomial model of pause occurrence in the first, pre-verbal position included significant effects of both output word order and information structure [*p* = 0.006, χ^2^ = 7.5 and *p* < 0.001, χ^2^ = 29.7, respectively]. It suggested that pauses preceding verbs were overall more frequent in SVO than in OVS word order [*estimate* = 0.531, *SE* = 0.1957, *z* = 2.7133, *p* < 0.01; positive estimates indicate more frequent pause occurrence]. Regarding information structure, the model indicated that these pauses were significantly less frequent in contrastive object focus (CFO) and significantly more frequent in narrow subject focus (NFS) compared to BF condition [*estimate* = −0.8460, *SE* = 0.2916, *z* = −2.9008, *p* = 0.004 and *estimate* = 0.8912, *SE* = 0.2590, *z* = 3.4412, *p* < 0.001, respectively]. The left panels of Figure 6 illustrate that this was true in both word orders.

With respect to pauses following the verb, the best model included interactions between information structure and word order [*p* < 0.001, χ^2^ = 43.7]. Main effects of information structure reflected the distribution in the intercept word order OVS, i.e., marginally fewer pauses in contrastive object focus (CFO) [*estimate* = −1.1328, *SE* = 0.5866, *z* = −1.9312, *p* = 0.053] and significantly fewer pauses in narrow object focus (NFO) [*estimate* = −1.5983, *SE* = 0.5214, *z* = −3.0653, *p* = 0.002] than in broad focus (see top right panel of Figure 6). In SVO word order, by contrast, post-verbal pauses were significantly more frequent in both object focus conditions (CFO and NFO) [*estimate* = 1.4826, *SE* = 0.6749, *z* = 2.1967, *p* = 0.028 and *estimate* = 2.1545, *SE* = 0.6362, *z* = 3.3866, *p* < 0.001, respectively], while being significantly less frequent in narrow subject focus (NFS) and marginally less frequent in contrastive subject focus (CFS) [*estimate* = −1.7060, *SE* = 0.5430, *z* = −3.1417, *p* = 0.002 and *estimate* = −0.9927, *SE* = 0.5470, *z* = −1.8150, *p* = 0.069, respectively], see bottom right panel in Figure 6. Thus, pauses after the verb occurred more often before a constituent in narrow focus in SVO word order. When the other, sentence-initial constituent was focused, pauses after the verb occurred less often in both word orders.

The analyses of pause occurrence were based on a native speaker's annotation, who inspected the waveforms and the spectrograms, and labeled all perceivable pauses irrespective of their duration (see Romøren and Chen, 2015, on advantages of this method over setting a minimum pause duration). The shortest annotated pause was a little over 1 ms long, while the longest was 458 ms, with a median of 9 ms and a standard deviation of 44 ms. An analysis of pause duration showed no significant effect of information structure [*p* = 0.548, χ^2^ = 3.1]. The best model for the duration of all 567 pauses realized by the participants also included an effect of pause position [*p* < 0.001, χ^2^ = 16.4], which indicated that pauses preceding the verb were overall significantly longer than those following the verb [*estimate* = −14.1695, *SE* = 3.4734, *t* = −4.0795, *p* < 0.001]. An alternative model additionally containing a marginal effect of word order, which indicated longer durations for pauses in SVO sentences, only provided a marginally better fit to the data [*p* = 0.076, χ^2^ = 3.1]. Modeling the duration of pauses preceding the verb separately, there was no significant effect of information structure [*p* = 0.626, χ^2^ = 2.6] and a model including word order was only marginally better than a null model without any predictor variables [*p* = 0.060, χ^2^ = 3.5]. For the subset of pauses following the verb, neither word order nor information structure had a significant effect [*p* = 0.592, χ^2^ = 0.3 and *p* = 0.954, χ^2^ = 0.7, respectively].

Thus, we found no relevant variation in pause duration, but a significant increase in the occurrence of pauses both before and after constituents in narrow focus for several conditions. Conversely, pauses occurred significantly less often before or after an unfocused constituent, i.e., when another constituent in the sentence was focused. In other words, the data indicated a tendency toward prosodic separation of focused constituents into prosodic phrases of their own, whereas non-focused constituents were more frequently not separated and rather phrased with the verb. Contrastive and narrow focus conditions generally displayed the same pattern, although effects did not always reach significance. Interestingly, a significant increase in pauses only appeared for SVO sentences, either separating initial subjects from the rest of the sentence with following pauses or disconnecting the final object and the preceding constituents. Recall that while sentences with unmarked SVO word order are by default divided into information structural fields as shown in Table 1, this word order is appropriate with other information structures as well. Participants might thus have used the insertion of pauses as part of a prosodic strategy for disambiguation.

### 3.5. Voice quality

We evaluated voice quality in a binary fashion here, determining for each syllable whether it was realized with modal voice throughout or whether it was realized (partially or completely) with non-modal voice, e.g., creaky, breathy or whispery voice. This annotation was based on waveform, spectrogram and auditory impression. Syllable-based evaluation was chosen as a compromise between a rather coarse word-by-word analysis and a detailed phoneme-level approach, which would make comparisons between different lexical items difficult. A binary syllable-level evaluation of voice quality has shown effects of information structure in adult Finnish, see Arnhold (2016) who also discusses differences between this method and a study on the effects of focus on voice quality in Finnish by Vainio et al. (2010) using inverse filtering (also see Epstein, 2002; Ní Chasaide et al., 2011, on English). Out of the 1136 words considered in the prosodic analyses, 1131 (nearly 100%) were disyllabic or longer and 536 (47%) had at least three syllables. We therefore restricted our analyses to voice quality of the first three syllables, discarding later syllables of longer words for the sake of comparability.

With the original coding of the data, some interactions could not be tested conclusively because more complex models failed to converge. The most complex converging model included an effect of syllable number indicating that second and third syllables were realized with non-modal voice quality more often than first syllables [*p* < 0.001, χ^2^ = 113.0; for second syllables: *estimate* = 0.88107, *SE* = 0.09880, *z* = 8.918, *p* < 0.001; for third syllables: *estimate* = 1.06692, *SE* = 0.12089, *z* = 8.825, *p* < 0.001]. It also contained significant interactions between word order and focus condition [*p* = 0.018, χ^2^ = 11.9] and between constituent and focus condition [*p* = 0.006, χ^2^ = 14.6]^6^. They indicated that there were less non-modal realizations in narrow subject focus overall compared to broad focus, but more in narrow subject focus for SVO sentences [*estimate* = −0.85446, *SE* = 0.24766, *z* = −3.450, *p* < 0.001 and *estimate* = 0.90481, *SE* = 0.27325, *z* = 3.311, *p* < 0.001, respectively] and that there were overall less non-modal realizations of subject than object nouns and less in contrastive subject focus than in broad focus, but significantly more non-modal realizations of subjects in contrastive subject focus condition [*estimate* = −0.82535, *SE* = 0.14079, *z* = −5.862, *p* < 0.001, *estimate* = −0.74142, *SE* = 0.24652, *z* = −3.008, *p* = 0.003 and *estimate* = 0.75097, *SE* = 0.25669, *z* = 2.926, *p* = 0.003, respectively]. When SVO word order was taken as the reference level instead of OVS, the interaction between word order and information structure showed a significant difference between broad focus and narrow object focus, indicating less non-modal realizations for OVS word order in narrow object focus [*estimate* = −0.90475, *SE* = 0.27321, *z* = −3.311, *p* < 0.001].

**Figure 7:**
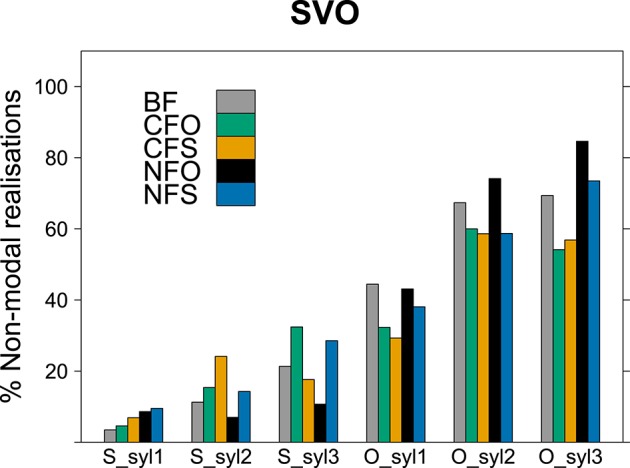
**Frequency of syllables with non-modal voice quality in SVO sentences (in %)**.

**Figure 8:**
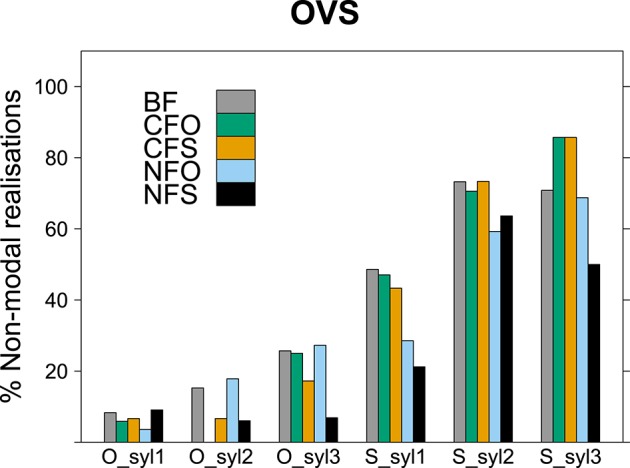
**Frequency of syllables with non-modal voice quality in OVS sentences (in %)**.

As illustrated in Figures 7, 8, the use of non-modal voice in final narrow focus (NFO in SVO order and NFS in OVS; black bars) deviated from all other information structures in both word orders (lighter bars). The figures show the percentage of syllables with non-modal voice in the different information structural conditions for output SVO and OVS order, respectively. The first group of bars illustrates the percentages for the first syllable of the sentence-initial word (subject for SVO; object for OVS) and the second and third group give percentages for the second and third syllable of the initial word. The three groups of bars on the right of each panel indicate the occurrence of non-modal voice for the first three syllables of the sentence-final word (object for SVO; subject for OVS).

A model comparing sentence-final narrow focus with all other information structural conditions provided an equally good or better fit to the data as models with the original coding of information structural conditions. This model found a significant interaction of information structure with syllable position [*p* = 0.024, χ^2^ = 13.0]. It indicated that creaky, breathy or whispery realizations were significantly more frequent for all later syllables than for first syllables of sentence-initial constituents, i.e., subjects in SVO and objects in OVS, respectively [for second syllables in initial words: *estimate* = 1.0294, *SE* = 0.2424, *z* = 4.2475, *p* < 0.001; for third syllables in initial words: *estimate* = 1.7515, *SE* = 0.2593, *z* = 6.7548, *p* < 0.001; for first syllables in final words: *estimate* = 2.5986, *SE* = 0.2258, *z* = 11.5097, *p* < 0.001; for second syllables in final words: *estimate* = 3.7753, *SE* = 0.2297, *z* = 16.4367, *p* < 0.001; for third syllables in final words: *estimate* = 3.8912, *SE* = 0.2551, *z* = 15.2514, *p* < 0.001]. As the figures demonstrate, voice quality was mostly modal early in the sentence, whereas the percentage of non-modal realizations rose steadily throughout the sentence in both word orders and for most information structural conditions. The model further indicated that in sentences with narrow focus on the final constituent (NFO in SVO order and NFS in OVS order), non-modal realizations were significantly less frequent on the second and third syllable of the sentence-initial word than in the other information structural conditions [second syllables: *estimate* = −1.3535, *SE* = 0.6274, *z* = −2.1573, *p* = 0.031; third syllables: *estimate* = −1.5827, *SE* = 0.6701, *z* = −2.3618, *p* = 0.018]. This reflects the deferred rise in the percentage of non-modal realizations for the NFO condition in SVO word order and for the NFS condition in OVS visible for the black bars in Figures 7, 8, respectively. Additionally, the model contained an interaction between information structure and word order [*p* = 0.014, χ^2^ = 6.0], suggesting that sentences with narrow focus on the final constituent displayed overall more non-modal realizations when the word order was SVO than when it was OVS [*estimate* = 0.7488, *SE* = 0.3092, *z* = 2.4218, *p* = 0.016], compare the last three black bars in Figures 7, 8.

To sum up, our analyses showed an interaction between word order and information structure: While the use of non-modal voice quality increased steadily throughout the sentence in most information structural conditions, a different pattern consistently emerged in conditions with non-contrastive final focus. In these conditions (NFO in SVO word order and NFS in OVS), non-modal voice remained infrequent throughout the first constituent and then increased abruptly on the final focused constituent. With the narrow focus occupying the final position, these utterances showed a perfect agreement between word order and information structure (recall Table 1). The prosodic realization underscored this agreement: Since non-modal voice quality is associated with finality in Finnish (Iivonen, 1998; Myers and Hansen, 2007; Nakai et al., 2009), avoiding non-modal voice quality earlier in the sentence but increasing its use on the focused constituent highlighted the fact that the focused constituent occupied the final position.

## 4. Discussion

Using a language game elicitation task, we found several significant effects of information structure on prosody, as well as some adjustments of word order, in the speech of Finnish 4- to 5-year-olds. The data showed both differences from and similarities with Finnish adults' marking of information structure as described in the literature. The next sections discuss this for our findings regarding word order, prosody, and the relationship between them, before Section 4.3 compares the present findings to research on children acquiring other languages.

Overall, an interesting observation is that the results frequently showed differences between contrastive and narrow focus conditions, with only one or the other showing significant differences from broad focus for word order, *f*_0_ range and voice quality. Different marking for contrastive and non-contrastive focus has been observed for several languages. For example, Northern Bizkaian Basque possesses prosodic means to mark contrastive focus that are not applied to mark non-contrastive narrow focus on the same word (Elordieta, 2008) and Catalan uses different pitch accents to mark contrastive and non-contrastive focus (Prieto, 2014). For adult Finnish, Arnhold (2016) observed the same prosodic strategies marking contrastive and non-contrastive focus, with larger effect sizes for contrastive focus. This contrasts with the present findings for child language. However, in terms of word order, the difference between contrastive and non-contrastive focus is firmly entrenched in adult Finnish, as detailed in the introduction.

### 4.1. Word order

Our participants mostly retained the word order provided in the input and only showed a significant effect of information structure in one condition, changing input OVS to SVO word order when the experimenter's question induced contrastive focus on the object. Most noticeably, the participants did not employ word order to mark contrast. They almost exclusively produced SVO and OVS word orders, which leave the sentence-initial contrast position unfilled. Thus, participants never used SOV word order in contrastive subject focus (or any other information structure condition) and only one sentence with OSV order appeared in contrastive object focus (with another one appearing in narrow object focus). This might indicate that at age five, Finnish children have yet to acquire the correspondence between word order and information structure shown in Table 1. At any rate, participants in the present study exhibited no signs of competence in the usage of the sentence-initial contrast position. Instead, the only significant change in word order moved the focused constituent into sentence-final condition, which might suggest that participants have over-generalized the function of the sentence-final focus position to accommodate both contrastive and narrow focus or indeed that it is reserved for contrastive foci in their grammar. This change did however lead to improved congruence between word order and information structure compared to the input, as discussed in Section 3.1. This suggests that children have acquired the relation between information structure and word order at least to a certain extent. Prosodic findings further strengthen this conclusion, as discussed below.

In fact, syntactic marking of contrast by using the contrast position is not obligatory in adult Finnish. Non-contrastive narrow focus can be marked by prosody alone in unmarked word order (Välimaa-Blum 1988, p. 75; Vainio and Järvikivi 2007). Also, contrastively focused subjects and objects can appear in SVO sentences with appropriate prosody, although it is not clear whether prosodic and word order marking are completely equivalent, especially for contrastive subjects (Heinämäki, 1982; Vallduví and Vilkuna, 1998; Kaiser, 2000; Molnár and Järventausta, 2003; Kaiser, 2006; Karlsson, 2008). At least in written language, use of the contrast position seems relatively rare: A corpus study of about 10,000 sentences found that 49% of the sentences had SVX order (where X stands for any non-subject NP, including objects), while SXV and XSV order occurred for only 1 and 3%, respectively (Hakulinen and Karlsson, 1995, p. 311). Finally, a corpus study by Kaiser (2000) found three discourse functions of OSV order. While marking the object as contrastive was a sub-case of the most common function appearing for 55% of OSV sentences, 36% of OSV sentences placed salient, but non-contrastive given information in initial position, while about 9% fronted new information.

Altogether, the use of the contrast position is thus a complex and non-obligatory strategy, making the fact that children did not employ it in the present study less surprising. By contrast, children's prevalent use of SVO word order in the present study is in line with the fact that Finnish is undoubtedly an SVO language both in terms of frequency and in terms of the acceptability of SVO in most information structural conditions. Further, while our participants only significantly adjusted input word order in one condition, this adjustment did improve the congruence between information structure and word order.

### 4.2. Prosody

With respect to prosody, the results of the present study evidenced effects of information structure on all investigated phonetic measures affected in adult speech, i.e., duration, pausing, voice quality and, to a limited degree, *f*_0_ range. These effects did not appear for all combinations of word order and information structural condition. However, where they did occur, they overwhelmingly reflected the prosodic focus marking strategies used by Finnish adults (e.g., Arnhold, 2016), suggesting that children possess some competence in the use of these strategies at age five. Where information structural effects appeared in our materials, *f*_0_ range was larger for the noun in contrastive focus than for the other noun in the sentence. Durational effects lead to shorter durations for unfocused, i.e., previously mentioned or given nouns. Preceding and following pauses were more frequent in several narrow focus conditions. At the same time, final narrow focused constituents showed an increased use of non-modal voice quality following an infrequent use of non-modal voice on the other, unfocused constituents preceding them in the same sentence. These effects were in congruence with Finnish adults' prosodic focus marking with one exception: Pauses preceding constituents in narrow focus have not been reported for adult Finnish, but are in line with other research on child language (see Section 4.3).

None of the prosodic parameters analyzed here displayed significant differences between all information structural conditions. However, all combinations of word order and information structure showed significant effects for at least one of the prosodic measures. For example, narrow subject focus in SVO sentences did not differ from broad focus in terms of *f*_0_ range, but did differ in terms of voice quality, while contrastive object focus in OVS sentences showed an indication of *f*_0_ range adjustments, but no effects on voice quality.

The distribution and nature of prosodic effects can be related to the interplay between word order and information structure by distinguishing three cases, discussed in detail below: matching word order and information structure, mismatch between word order and information structure, and unmarked word order with non-default information structure. Only duration displayed the same pattern across both word orders produced by the children: Durations of object nouns were significantly shorter in subject focus conditions—both contrastive and narrow—compared to broad focus. This can be interpreted as an effect of givenness, shortening the unfocused word, or possibly as an indirect way of making the focused subject constituent appear relatively longer. The other phonetic measures showed an influence of both word order and information structure. Interestingly, a division of labor appeared between the different measures; while some underscored a congruence between word order and information structure, some compensated for mismatches and some disambiguated unmarked cases.

#### 4.2.1. Matching word order and information structure

The default interpretation of the SVO order predominantly used by the children is that the subject occupying the topic position is a topic and/or mentioned in the previous discourse (given) while the sentence-final object is in non-contrastive focus and/or new, see (a) in Table 3. For OVS, correspondingly, objects are usually given while subjects are focused, see (b). Thus, these word orders perfectly matched the information structure induced by the experimenter's questions in narrow object focus (NFO) and narrow subject focus condition (NFS), respectively. In precisely these two conditions, significant effects appeared for voice quality, with a delayed onset of sentence-final non-modal voice highlighting the finality of the focused constituent and thus the match between word order and information structure (see Section 3.5). Otherwise, sentences in these conditions did not differ much from broad focus realizations, in line with an information structure with final narrow focus being the default interpretation. Heinämäki (1982) in fact argues that both SVO and OVS are default word orders in Finnish, but it is noteworthy that SVO sentences additionally showed a significant increase in the use of pauses before final narrowly focused objects.

**Table 3:** **Discourse configurational analysis for conditions with word order matching information structure**.

	**Contrast**	**Topic**	**Rest (focus final)**
(a)		S	V[O]_NF_
(b)		O	V[S]_NF_

#### 4.2.2. Unmarked word order and information structure

While a perfect match between word order and information structure appears in (a) in Table 3, unmarked SVO word order may appear felicitously with other information structures. In addition to broad focus, participants also produced SVO word order with contrastive and non-contrastive subject focus, as well as with the object in contrastive focus (conditions CFS, NFS, and CFO, respectively). Two of these conditions, NFS and CFO, were realized with significantly more pauses preceding or following the constituent in narrow focus, while the third showed an insignificant increase in the occurrence of pauses compared to the broad focus condition. In sentences with unmarked word order, the use of pauses thus frequently distinguished information structural conditions.

#### 4.2.3. Mismatch between word order and information structure

One of the most striking findings of the present study was that *f*_0_ contours of SVO sentences showed no significant differences between information structural conditions. Changes in *f*_0_ range are a well-established part of prosodic focus marking in Finnish (e.g., Välimaa-Blum, 1993; Mixdorff et al., 2002; Suomi et al., 2003) and should be expected especially in the default word order, i.e., in the absence of syntactic marking of information structure (Arnhold and Féry, 2013). However, as mentioned in the introduction and discussed further in Section 4.3, children frequently underuse *f*_0_ as a cue to information structure while overusing other prosodic cues like pauses compared to adults. In the present data, there was only limited evidence for significant adjustments of *f*_0_ range in two conditions, OVS sentences with contrastive focus on the subject (CFS) and OVS sentences with contrastive focus on the object (CFO). As illustrated in (a) in Table 4, productions of OVS orders in CFO condition showed a mismatch between the information structure implied by the use of the more marked word order and the information structure induced by the experimenter's question. Although participants frequently avoided this mismatching constellation by changing input word order, they were overall likely to retain the input word order. By expanding the *f*_0_ range of the contrastively focused constituent and compressing the *f*_0_ range of the other noun in the sentence, the prosody of children's productions compensated for this mismatch when they did produce it.

**Table 4:** **Discourse configurational analysis for conditions with mismatch between word order and information structure**.

	**Contrast**	**Topic**	**Rest (focus final)**
(a)		[O]_CF_	VS
(b)		O	V[S]_CF_
(c)		[O]_NF_	VS

The other constellation with an indication of significant *f*_0_ range adjustments, CFS with OVS word order, appears in (b) in Table 4. In this condition, participants made the focused subject more prominent by expanding its *f*_0_ range. Recall that children practically never placed a constituent in the sentence-initial contrast position and may not have had acquired its use yet (cf. Section 4.1). Therefore, it is somewhat unclear whether the constellation in (b) in Table 4 constitutes a (slight) mismatch in their grammar—as the position for non-contrastive foci contains a contrastive focus–or whether it is instead a perfect match—as the focused constituent occupies the only focus position they have acquired at this point. Interestingly, while children may have adjusted *f*_0_ range for this constellation, they did not do so for the mirror image SV[O]_CF_ (i.e., CFO with SVO order). This is in line with adult intuitions that while contrastive objects are acceptable in the sentence-final position, contrastive subjects are not (Kaiser, 2000).

Finally, in a further mismatching condition, OVS sentences with narrow object focus (NFO; see (c) in Table 4), another type of prosodic compensation appeared. Here, the frequency of pauses preceding the subject decreased significantly, prosodically grouping the subject with the preceding verb. Recall that by contrast, focused constituents were more frequently separated from the verb by a pause.

Overall, our data suggest that, like Finnish adults (Vainio and Järvikivi, 2007), 4- to 5-year-olds are able to apply prosodic compensation for a mismatch between word order and information structure. This suggests that although participants rarely changed the input word order, they demonstrated competence regarding the correspondence between word order and information structure. Further, the findings indicated that children are already able to bring together two areas of grammar, word order and prosody, setting them on the path to acquiring information structure marking as a complex system of interacting strategies.

### 4.3. Comparison with children acquiring other languages

The present data did not display the tendency toward a new-before-given word order frequently reported for child speech (Narasimhan and Dimroth, 2008). This can in part be explained by the task, since the input word order, which significantly influenced children's production, was perfectly balanced between given-before-new and new-before-given. However, it is noteworthy that children's only significant deviation from the input placed the narrowly focused constituent in final position, resulting in a given-before-new order. This finding was not only in accordance with adult Finnish grammar, as argued above, but also contradicts a universal tendency for children of the age range under investigation to prefer new-before-given (note that Narasimhan and Dimroth, 2008, review other studies contradicting this tendency).

Regarding task effects, the study by Sauermann et al. (2011), investigating German-learning 4-year-olds, provides an interesting opportunity for comparison with the present one. Using the same design as the current study, they, too, found an effect of input word order. As in the present experiment, significant differences between information structural conditions appeared only with the more marked OVS input, but in contrast to the present results, these effects did not mirror information structure effects reported in the literature on adult German (the adult control group consistently used unmarked SVO order in all conditions). Instead, children retained input OVS order most frequently in broad focus, i.e., in an information structural condition when it is highly inappropriate in adult German, which the authors explain with reference to memory constraints. Chen and Höhle (submitted) report the same finding for Dutch 4- to 5-year-olds. Thus, these results suggest that children acquiring West Germanic languages have limited competence regarding the connection between information structure and word order, but are more influenced by general cognitive factors. In comparison, the finding of a significant information structural effect on word order in the present study seems all the more remarkable.

With respect to prosody, the current results mirror existing studies in showing that 4- to 5-year-olds use prosody to mark information structure, but are not yet employing it in a completely adult-like manner (e.g., Wells et al., 2004; Chen, 2007, 2009, 2011). More specifically, our participants deviated from adult speech through incomplete application of prosodic focus marking strategies. The only exception concerns the use of pauses. Here, children displayed a focus marking strategy not reported for adult speakers of Finnish by showing an increased frequency of pauses not only after words in (non-contrastive or contrastive) narrow focus, but also preceding them. While children generally pause more frequently than adults (e.g., Redford, 2013), the present data showed significant effects of information structure, with the use of pauses increasing specifically in the context of narrow focus. Interestingly, this finding is reminiscent of Romøren and Chen's (2015) observation that Dutch 4- to 5-year-olds showed longer pause durations before narrow focus constituents, while the same strategy was less prevalent in adult speech. Their hypothesis that the extended use of pausing is related to the reduced use of other prosodic markers fits with the current data set as well: Just as Dutch children show incomplete mastery of accentuation, participants in the present study underused the prosodic focus markers employed by Finnish adults, especially *f*_0_ range. In adult Finnish, overall significant effects appear for all the prosodic measures investigated here. By contrast, in the present study none of measures showed significant distinctions between all three basic information structural conditions, i.e., broad focus, narrow focus and givenness, for both constituents and in both word orders— although all measures did show some significant differences. Thus, children seemed to have acquired all prosodic focus markers, but did not use any of them as prevalently as adults.

Most notably, our participants did not employ *f*_0_ range adjustments in unmarked SVO order, but reserved the use of *f*_0_ for range adjustments in unmarked SVO order, but reserved the for contrastive focus in the more marked OVS word order. This finding is not only in contrast to research on adult Finnish, but also differs from results for Dutch and German children completing the same task. In direct opposition to our Finnish findings, Dutch 4- to 5-year-olds employed *f*_0_ adjustments only in unmarked word order (Chen and Höhle, submitted), whereas German children showed *f*_0_ effects in both word orders (Sauermann et al., 2011). Based on the relative prevalence of prosodic vs. word order effects of information structure in their data, Sauermann et al. (2011) suggest that children may prefer prosodic strategies due to their comparative simplicity. The results of the current study indicate that this analysis is not suitable for Finnish, not only because a significant word order effect of information structure appeared, but also more crucially because prosodic effects were consistently modulated by the (lack of) congruence between information structure and word order.

In German-speaking children, significant prosodic effects only appeared for subject nouns (Sauermann et al., 2011). A somewhat parallel finding of the present study was the restriction of durational effects to object nouns in both word orders. Otherwise, however, prosodic effects never emerged only for subjects or only for object nouns in principle. Instead, significant differences either appeared for subjects and objects equally (*f*_0_ range in contrastive focus, see Section 3.2) or were restricted to either subject or object nouns in specific combinations of information structural conditions and word order. For example, an increased frequency of realizations with non-modal voice quality appeared for narrowly focused objects in SVO sentences, but not for the subjects, and for narrowly focused subjects, but not objects, in OVS sentences (see Section 3.5). As argued in the previous sections, the distribution of the presence or absence of prosodic effects suggests that Finnish 4- to 5-year-olds have already partially acquired the correspondence between word order and information structure in Finnish, as well as interactions between prosody and word order. This contrasts with children acquiring Germanic languages who showed clearer differences between grammatical functions, i.e., subjects differing from objects in the same way for SVO and OVS word order. This observation may be related to the principled differences between the languages. In German, word order variation is a possible tool for marking information structure, although prosodic marking is more prevalent. In Finnish, word order is largely determined by information structure (Vilkuna, 1989, 1995), and word order and prosody interact and can compensate each other in marking information structure (Vainio and Järvikivi, 2007; Arnhold and Féry, 2013). The comparison between our results and findings of previous studies on a Germanic language applying the same method suggests that this difference in grammatical organization is also reflected in acquisition: At age four to five, children acquiring a discourse configurational Uralic language showed a complex interplay between prosody and word order, suggesting a more central role of word order than for children acquiring a Germanic language. Thus, cross-linguistic differences in grammatical organization and functional weight are reflected in language development. Our results further underline the importance of representing a broad sample of languages when investigating the acquisition of prosody as well as other aspects of grammar.

## 5. Conclusions

Based on a semi-spontaneous production experiment, the present study indicated that Finnish 4- to 5-year-olds use both prosody and word order to mark information structure. Although the use of syntactic markers and prosodic markers was very limited compared to adult speech, significant effects appeared for nearly all prosodic parameters reported to mark focus in Finnish adults (the exception being intensity, which we did not investigate due to the nature of the data).

Crucially, prosodic marking interacted with word order: Differing from adult Finnish, significant adjustments of *f*_0_ only appeared when compensating a mismatch between word order and information structure. By contrast, a distinct pattern in the use of non-modal voice quality, also found for adult speakers, highlighted perfect matches between word order and information structure. A significant increase in the use of pauses before and/or after constituents in narrow focus only appeared in sentences with unmarked SVO word order, while adjustments in word duration appeared for both word orders, but affected only object nouns.

The modulation of prosodic effects by word order reflects the central role of the relationships between information structure, word order and prosody in Finnish, contrasting with the development of children acquiring Germanic languages, who predominantly show purely prosodic information structure marking at the same age.

## Author contributions

AC and JJ constructed the experiment and created materials. JJ oversaw running the experiment. AA analyzed the data. AA, AC, and JJ wrote the paper.

## Funding

AC was supported by a VIDI grant from the Netherlands Organization for Scientific Research (NWO-276-89-001) during the writing of the manuscript.

### Conflict of interest statement

The authors declare that the research was conducted in the absence of any commercial or financial relationships that could be construed as a potential conflict of interest.

## Footnotes

^1^For comparability with the present study, this overview concentrates on language production; on children's sensitivity to information structure and its marking in perception, see e.g. Ito and Speer (2008); Fernald et al. (2010); Ito et al. (2014); Järvikivi et al. (2014); see Chen (2010) for evidence that 4- to 5-year-olds possess similar skills in production and perception of prosodic focus marking.

^2^These terms somewhat simplify Vilkuna's description of the correspondence between information structural roles and positions. For generative accounts of Finnish discourse configurationality, also see Holmberg et al. (1993); Kaiser (2000, 2006); Holmberg and Nikanne (2002); Molnár and Järventausta (2003).

^3^Note that Vilkuna (1989, 1995) and Vallduví and Vilkuna (1998) do not employ the alternatives-based definition of focus adopted here, but a newness-based definition of focus (and topic). This difference is not relevant here and will be ignored in the following, since these dimensions were correlated in the present data.

^4^Conditions were not distributed exactly equally in the removed items, but there were no large differences either. Of removed sentences with SVO input word order, 18 had BF as the intended information structure, 25 CFO, 26 CFS, 20 NFO, and 18 NFS. Among removed sentences with input OVS order, 22 were intended BF, 20 CFO, 13 CFS, 21 NFO, and 14 NFS.

^5^Significant main effects of input word order [*estimate* = 6.4843, *SE* = 1.1024, *z* = 5.882, *p* < 0.001] and information structure [*estimate* = 1.7947, *SE* = 0.4841, *z* = 3.707, *p* < 0.001] appeared, while the interaction between them was marginal [*p* < 0.080, χ^2^ = 3.1; *estimate* = −2.9450, *SE* = 1.5258, *z* = −1.930, *p* = 0.054]. The interaction appeared significant when a by-participant random effect of information structure was included in the model [*estimate* = −3.3543, *SE* = 1.6483, *z* = −2.035, *p* = 0.042], but this did not significantly improve model fit [*p* = 0.755, χ^2^ = 0.6].

^6^Separate comparisons with a simple model also indicated a significant interaction between constituent and word order [*p* < 0.001, χ^2^ = 655.6], suggesting that non-modal voice quality was generally more frequent later in the sentence, i.e., more non-modal voice for subjects than for objects and more non-modal voice in SVO order than in OVS, but less non-modal voice for subjects in SVO sentences, where they were in initial position [*estimate* = 2.8597, *SE* = 0.1978, *z* = 14.454, *p* < 0.001, *estimate* = 2.6861, *SE* = 0.1808, *z* = 14.858, *p* < 0.001 and *estimate* = −5.4059, *SE* = 0.2472, *z* = −21.871, *p* < 0.001, respectively]. However, a model adding this interaction to the other two did not converge anymore, suggesting that model complexity was too high for the amount of data. To assess interactions between sentence position and focus condition, we therefore report the results of modeling with a simplified coding below.
