# Supplementary material for: Acquiring Complex Focus-Marking: Finnish 4- to 5-Year-Olds Use Prosody and Word Order in Interaction
Source: Front Psychol. 2016 Dec 1;7:1886. doi: 10.3389/fpsyg.2016.01886 (PMC5131328; doi:10.3389/fpsyg.2016.01886)
Supplement: Supplementary file 1 [file Table1.pdf]

Supplementary Material: Acquiring complex focus marking: Finnish four- to five-year-olds use prosody and word order in interaction

Anja Arnhold [1,2,*], Aoju Chen [3] and Juhani Järvikivi [2]
*Correspondence:
Anja Arnhold
anja.arnhold@gmail.com

1 STIMULUS MATERIALS

The table below lists the experimenter's introduction of the stimulus picture, the experimenter's question prompt and the robot's answer for all practice and target trials of an experimental session. The experimenter's question prompt for the participant was exactly the same as her question to the robot in all cases except one. For last practice trial, the prompt question "What is happening?" was followed by "How do we say that in human language?" only when addressing the participant, but not when addressing the robot.

The table additionally classifies the experimental conditions, i.e. word order in the robot's answer (input word order) and information structure introduced for the target utterance. Target trials are shown in one of the two presentation orders; half of the participants encountered them in reversed order. Note that the word order of the robot's answers is not indicated in the English translations and that the translations use progressive tense, whereas the Finnish prompts did not. Furthermore, the translations include articles (definite or indefinite as contextually appropriate), whereas standard Finnish does not have articles.

Supplementary Table | Trial number (Trial), information structural condition (IS), input word order (WO), experimenter's introduction of the picture, experimenter's question prompt and robot's answer for all practice and target trials of an experimental session.

Trial	IS	WO	Introduction	Question prompt	Robot's answer
Practice	CFO	SVO	*Tässä kuvassa on mies. Mies näyttää rakentavan jotain.*	*Rakentaako mies laivaa?*	*Mies rakentaa taloa.*
			"In this picture, there is a man. The man looks like he is building something."	"Is the man building a boat?"	"The man is building a house."
Practice	NFS	OVS	*Tässä kuvassa joku näyttää syövän porkkanaa.*	*Kuka syö porkkanaa?*	*Porkkanaa syö jänis.*
			"In this picture, it looks like someone is eating a carrot."	"Who is eating a carrot?"	"A hare is eating the carrot."

Supplementary Table (continued).

Trial	IS	WO	Introduction	Question prompt	Robot's answer
Practice	BF	SOV	*Tässä kuvassa on talo, mutta se, mitä kuvassa tapahtuu, on peitetty kokonaan.*	*Mitä kuvassa tapahtuu?*	*Poika tyttöä kiusaa.*
			"In this picture, there is a house but that which is happening in the picture is completely covered."	"What is happening in the picture?"	"A boy is teasing a girl."
1	BF	SVO	*Tässä kuva on taas kokonaan peitetty.*	*Mitä tässä tapahtuu?*	*Haltija maalaa maljakkoa.*
			"Here, the picture is again covered completely."	"What is happening here?"	"An elf is painting a vase."
2	NFO	OVS	*Tässä on sammakko. Sammakko näyttää nielaisevan jotain.*	*Mitä sammakko nielaisee?*	*Pallon nielaisee sammakko.*
			"Here is a frog. The frog looks like it is swallowing something."	"What is the frog swallowing?"	"The frog is swallowing a ball."
3	CFS	SVO	*Joku näyttäisi rakentavan muuria.*	*Rakentaako mies muuria?*	*Tyttö rakentaa muuria.*
			"It looks like someone is building a wall."	"Is a man building the wall?"	"A girl is building the wall."
4	CFO	SVO	*Mehiläinen näkee jotain.*	*Näkeekö mehiläinen talon?*	*Mehiläinen näkee kukan.*
			"A bee is seeing something."	"Is the bee seeing a house?"	"A bee is seeing a flower."
5	BF	OVS	*Tämä kuva on taas peitetty.*	*Mitä tässä tapahtuu?*	*Ruohoa polkee varsa.*
			"This picture is covered again."	"What is happening here?"	"A foal is trampling grass."
6	NFS	SVO	*Joku pitelee kakkua.*	*Kuka pitää kakkua?*	*Tyttö pitää kakkua.*
			"Someone is holding a cake."	"Who is holding the cake?"	"A girl is holding the cake."
7	NFO	OVS	*Tässä on opettaja. Opettaja osoittaa karttakepillä jotain.*	*Mitä opettaja osoittaa?*	*Maljakkoa osoittaa opettaja.*
			"Here is a teacher. The teacher is showing something with a pointer."	"What is the teacher showing?"	"The teacher is showing a vase."
8	BF	OVS	*Kuva on taas peitetty.*	*Mitä tässä tapahtuu?*	*Lankakerän ottaa kiinni vauva.*
			"The picture is covered again."	"What is happening here?"	"A baby is catching / seizing a ball of wool."

Supplementary Table (continued).

Trial	IS	WO	Introduction	Question prompt	Robot's answer
9	CFS	SVO	*Joku työntää autoa.*	*Työntääkö koppakuoriainen autoa?*	*Kameli työntää autoa.*
			"Someone is pushing a car."	"Is a beetle pushing the car?"	"A camel is pushing the car."
10	CFO	OVS	*Lehmä näyttää syövän jotain.*	*Syökö lehmä pizzaa?*	*Kukkaa syö lehmä.*
			"A cow looks like it is eating something."	"Is the cow eating pizza?"	"The cow is eating a flower."
11	NFO	SVO	*Possu pesee jotain ammeessa.*	*Mitä possu pesee?*	*Possu pesee paitaa.*
			"A piggy is washing something in a tub."	"What is the piggy washing?"	"The piggy is washing a shirt."
12	CFO	OVS	*Tässä kuvassa on sammakko. Sammakko näyttää löytävän jotain.*	*Löytääkö sammakko kaapin?*	*Pallon löytää sammakko.*
			"In this picture, there is a frog. The frog looks like it is finding something."	"Is the frog finding a closet?"	"The frog is finding a ball."
13	BF	OVS	*Nyt kuva on taas peitetty niin, ettei näe, mitä siinä tapahtuu.*	*Mitä tässä tapahtuu?*	*Pallon ojentaa poliisi.*
			"Now the picture is again covered so that we cannot see what is happening there."	"What is happening here?"	"A police officer is giving out a ball."
14	NFO	SVO	*Kuvassa on hirviö. Hirviö heittää jotain.*	*Mitä hirviö heittää?*	*Hirviö heittää maljakkoa.*
			"There is a monster in the picture. The monster is throwing something."	"What is the monster throwing?"	"The monster is throwing a vase."
15	BF	SVO	*Kuva on taas peitetty.*	*Mitä tässä tapahtuu?*	*Vauva haluaa pullon.*
			"The picture is covered again."	"What is happening here?"	"A baby wants a bottle."
16	NFS	OVS	*Joku lukee lehteä.*	*Kuka lukee lehteä?*	*Lehteä lukee tyttö.*
			"Someone is reading a newspaper."	"Who is reading a newspaper?"	"A girl is reading the newspaper."
17	BF	SVO	*Kuva on peitetty.*	*Mitä tässä tapahtuu?*	*Varsa etsii heinää.*
			"The picture is covered."	"What is happening here?"	"A foal is looking for hay."
18	CFS	OVS	*Joku avaa ovea.*	*Avaako poika ovea?*	*Ovea avaa tyttö.*
			"Someone is opening a door."	"Is a boy opening the door?"	"A girl is opening the door."
19	NFS	OVS	*Joku juo maitoa pillillä.*	*Kuka juo maitoa?*	*Maitoa juo kameli.*
			"Someone is drinking milk with a straw."	"Who is drinking milk?"	"A camel is drinking milk."

Supplementary Table (continued).

Trial	IS	WO	Introduction	Question prompt	Robot's answer
20	CFO	SVO	*Pulu näyttää juonittelevan jotain.*<br>"A pigeon looks like it is plotting something."	*Varastaako pulu bussin?*<br>"Is the pigeon stealing a bus?"	*Pulu varastaa pallon.*<br>"The pigeon is stealing a ball."
21	BF	OVS	*Kuva on taas peitetty.*<br>"The picture is covered again."	*Mitä tässä tapahtuu?*<br>"What is happening here?"	*Leipää piilottaa kameli.*<br>"A camel is hiding a bread."
22	NFS	SVO	*Joku kampaa nukkea.*<br>"Someone is combing a doll."	*Kuka kampaa nukkea?*<br>"Who is combing the doll?"	*Lapsi kampaa nukkea.*<br>"A child is combing the doll."
23	CFS	OVS	*Joku kaivaa koloa maahan.*<br>"Someone is digging a hole into the ground."	*Kaivaako tiikeri koloa?*<br>"Is a tiger digging the hole?"	*Koloa kaivaa varsa.*<br>"A foal is digging the hole."
24	BF	SVO	*Kuva on taas peitetty.*<br>"The picture is covered again."	*Mitä tässä tapahtuu?*<br>"What is happening here?"	*Apina keittää kukkaa.*<br>"A monkey is cooking a flower."
25	CFO	OVS	*Tässä kuvassa on noita. Noita ompelee jotain.*<br>"In this picture, there is a witch. The witch is sewing something."	*Ompeleeko noita nallea?*<br>"Is the witch sewing a teddy bear?"	*Paitaa ompelee noita.*<br>"The witch is sewing a shirt."
26	BF	SVO	*Kuva on taas peitetty.*<br>"The picture is covered again."	*Mitä tässä tapahtuu?*<br>"What is happening here?"	*Kameli nuolee suolaa.*<br>"A camel is licking salt."
27	NFS	SVO	*Joku pitää lakkia.*<br>"Someone is holding a cap."	*Kuka pitää lakkia?*<br>"Who is holding the cap?"	*Varsa pitää lakkia.*<br>"A foal is holding the cap."
28	CFS	OVS	*Joku ottaa banaanin maasta.*<br>"Somebody is picking up a banana from the ground."	*Ottaako lääkäri banaanin?*<br>"Is a doctor picking up the banana?"	*Banaanin ottaa vauva.*<br>"A baby is picking up the banana."
29	NFO	OVS	*Hevonen näyttäisi polkevan jotain.*<br>"A horse looks like it is trampling something."	*Mitä hevonen polkee?*<br>"What is the horse trampling?"	*Kukkaa polkee hevonen.*<br>"The horse is trampling a flower."
30	BF	SVO	*Kuva on taas peitetty niin, ettei näe, mitä siinä tapahtuu.*<br>"The picture is again covered so that we cannot see what is happening there."	*Mitä tässä tapahtuu?*<br>"What is happening here?"	*Tyttö värjää paitaa.*<br>"A girl is coloring a shirt."

Supplementary Table (continued).

Trial	IS	WO	Introduction	Question prompt	Robot's answer
31	NFS	SVO	*Joku raaputtaa ikkunaa.*	*Kuka raaputtaa ikkunaa?*	*Kameli raaputtaa ikkunaa.*
			"Someone is scratching a window."	"Who is scratching the window?"	"A camel is scratching the window."
32	BF	OVS	*Kuva on taas peitetty kokonaan.*	*Mitä tässä tapahtuu?*	*Maljakkoa liimaa lääkäri.*
			"The picture is again covered completely."	"What is happening here?"	"A doctor is glueing a vase."
33	CFS	SVO	*Joku heiluttaa helistintä.*	*Heiluttaako vaari helistintä?*	*Vauva heiluttaa helistintä.*
			"Someone is waving a rattle."	"Is a grandfather waving the rattle?"	"A baby is waving the rattle."
34	BF	OVS	*Nyt ei taas näe, mitä kuvassa tapahtuu.*	*Mitä tässä tapahtuu?*	*Kukkaa haistaa hiiri.*
			"Now we can again not see what is happening in the picture."	"What is happening here?"	"A mouse is sniffing a flower."
35	NFO	SVO	*Tässä on isoäiti. Isoäiti näyttäisi antavan jotain kädestään.*	*Mitä isoäiti antaa?*	*Isoäiti antaa pallon.*
			"Here is a grandmother. The grandmother looks like she is giving something that is in her hand."	"What is the grandmother giving?"	"The grandmother is giving a ball."
36	BF	OVS	*Tässä on taas kuva kokonaan peitetty.*	*Mitä tässä tapahtuu?*	*Nurmikkoa leikkaa tyttö.*
			"Here again the picture is covered completely."	"What is happening here?"	"A girl is mowing a lawn."
37	CFO	SVO	*Tässä on kummitus. Kummitus näyttää repivän jotain.*	*Repiikö kummitus kirjaa?*	*Kummitus repii housuja.*
			"Here is a ghost. The ghost looks like it is ripping something [up]."	"Is the ghost ripping [up] a book?"	"The ghost is ripping [up] pants."
38	BF	SVO	*Kuva on taas kokonaan peitetty.*	*Mitä tässä tapahtuu?*	*Kirahvi huomaa pallon.*
			"The picture is again covered completely."	"What is happening here?"	"A giraffe is noticing a ball."
39	CFS	OVS	*Joku näyttäisi vetävän autoa perässään.*	*Vetääkö kala autoa?*	*Autoa vetää kameli.*
			"It looks like someone is pulling a car by its rear."	"Is a fish pulling the car?"	"A camel is pulling the car."
40	NFS	OVS	*Joku pitää haarukkaa kädessään.*	*Kuka pitää haarukkaa?*	*Haarukkaa pitää lapsi.*
			"Someone is holding a fork in their hand."	"Who is holding the fork?"	"A child is holding the fork."

Supplementary Table (continued).

Trial	IS	WO	Introduction	Question prompt	Robot's answer
41	NFO	SVO	*Jänis hautaa jotain maahan.*	*Mitä jänis hautaa?*	*Jänis hautaa kukkaa.*
			"A hare is burrying something in the ground."	"What is the hare burrying?"	"The hare is burrying a flower."
42	BF	OVS	*Tässä on kasa vaatteita, mutta ei näy, mitä kuvassa tapahtuu.*	*Mitä tässä tapahtuu?*	*Paitaa silittää poika.*
			"Here is a pile of clothes but we cannot see what is happening in this picture."	"What is happening here?"	"A boy is ironing a shirt."
43	NFS	OVS	*Tässä on tomaatti. Joku näyttäisi polkevan tomaattia.*	*Kuka polkee tomaattia?*	*Tomaattia polkee varsa.*
			"Here is a tomato. It looks like someone is trampling the tomato."	"Who is trampling the tomato?"	"A foal is trampling the tomato."
44	BF	SVO	*Kuva on taas peitetty kokonaan.*	*Mitä tässä tapahtuu?*	*Tyttö lakaisee katua.*
			"The picture is again covered completely."	"What is happening here?"	"A girl is sweeping a street."
45	CFO	SVO	*Prinsessa. Hän näyttäisi pesevän jotain.*	*Peseekö prinsessa autoa?*	*Prinsessa pesee maljakkoa.*
			"A princess. She looks like she is washing something."	"Is the princess washing a car?"	"The princess is washing a vase."
46	NFO	OVS	*Tässä on lapsi. Lapsi on ostamassa jotain.*	*Mitä lapsi ostaa?*	*Paitaa ostaa lapsi.*
			Here is a child. The child is buying something.	"What is the child buying?"	"The child is buying a shirt."
47	CFS	SVO	*Joku poimii mansikkaa.*	*Poimiiko leijona mansikkaa?*	*Varsa poimii mansikkaa.*
			"Someone is picking a strawberry."	"Is a lion picking the strawberry?"	"A foal is picking the strawberry."
48	CFO	OVS	*Tässä on kuningatar. Kuningatar saa lahjan.*	*Saako kuningatar jäätelön?*	*Maljakon saa kuningatar.*
			"Here is a queen. The queen is getting a gift."	"Is the queen getting an ice cream?"	"The queen is getting a vase."
